# Supplementary material for: ECOLE: Learning to call copy number variants on whole exome sequencing data
Source: Nat Commun. 2024 Jan 2;15:132. doi: 10.1038/s41467-023-44116-y (PMC10762021; doi:10.1038/s41467-023-44116-y)
Supplement: Supplementary file 1 — Supplementary Information [file 41467_2023_44116_MOESM1_ESM.pdf]

Supplementary Material
for
ECOLE: Learning to call copy number variants on whole exome sequencing data

1 Supplementary Tables

Supplementary Table 1. The performance comparison of the WES-based CNV callers on the 1000 Genomes data set (test set). CNVnator calls on the matched WGS samples are used as the ground truth. CNVkit and Control-FREEC return exact (integer) copy number predictions, which are discretized into deletion, duplication, and no-call. We also used the DECoNT tool to polish call sets of all considered tools which are denoted by DECONT-*tool_name*. Bold indicates the best result in that category.

**TOOLS**	**DEL Precision**	**DUP Precision**	**Overall Precision**	**DEL Recall**	**DUP Recall**	**Overall Recall**	**DEL F1 Score**	**DUP F1 Score**	**Overall F1 Score**
Control-FREEC	0.339	0.332	0.336	0.472	0.084	0.276	0.394	0.135	0.303
CNVkit	0.340	0.336	0.338	0.321	0.092	0.206	0.330	0.144	0.255
XHMM	0.407	0.434	0.421	0.080	0.071	0.076	0.134	0.122	0.129
CONIFER	0.317	0.105	0.211	0.004	0.007	0.006	0.008	0.013	0.012
CODEX2	0.007	0.009	0.008	0.187	0.157	0.172	0.013	0.017	0.015
GATK	0.013	0.028	0.021	0.074	0.140	0.107	0.023	0.046	0.035
DECoNT-Control-FREEC	0.341	0.414	0.371	**0.621**	0.049	0.335	0.440	0.087	0.352
DECoNT-CNVkit	0.325	0.338	0.332	0.160	0.171	0.165	0.214	0.227	0.220
DECoNT-XHMM	0.615	0.652	0.634	0.086	0.075	0.081	0.151	0.135	0.144
DECoNT-CONIFER	0.203	0.152	0.178	0.011	0.003	0.007	0.021	0.006	0.013
DECoNT-CODEX2	0.009	0.013	0.011	0.074	0.085	0.080	0.016	0.023	0.019
ECOLE	**0.834**	**0.703**	**0.769**	**0.531**	**0.470**	**0.501**	**0.649**	**0.563**	**0.606**

Supplementary Table 2. Confusion Matrices for the performance comparison based on CNVnator calls used as the ground truth for the 1000 Genomes data set (test set). These produce the precision and recall results in Supplementary Table 1.

		**Ground Truth**		
TOOLS	**Predicted**	NO CALL	DUP	DEL
Control-FREEC	NO CALL	7395972	6874622	7058879
	DUP	1324171	1284308	1248352
	DEL	7542829	6966242	7455429
DECoNT-Control-FREEC	NO CALL	5784309	5378215	5518070
	DUP	589108	734984	450704
	DEL	9889555	9011973	9793886
CNVkit	NO CALL	2047096	1938886	1921362
	DUP	299837	296390	284186
	DEL	1049869	974144	1043521
DECoNT-CNVkit	NO CALL	2290912	2124041	2205063
	DUP	557237	550779	521755
	DEL	548653	534600	522251
XHMM	NO CALL	47451741	417408	302298
	DUP	38137	32298	3916
	DEL	33510	4648	26294
DECoNT-XHMM	NO CALL	47489289	419118	303044
	DUP	17296	34214	953
	DEL	16803	1022	28511
CONIFER	NO CALL	15551274	174086	107047
	DUP	9484	1263	1280
	DEL	639	267	422
DECoNT-CONIFER	NO CALL	15554000	174200	107128
	DUP	3323	666	391
	DEL	4074	750	1230
CODEX2	NO CALL	31430578	300514	218459
	DUP	7445915	71440	51836
	DEL	8646895	82400	62213
DECoNT-CODEX2	NO CALL	41897336	395586	283708
	DUP	2834559	38655	24316
	DEL	2791493	20113	24484
GATK	NO CALL	27352941	226073	181310
	DUP	1369519	39463	13770
	DEL	1123082	17162	15517
ECOLE	NO CALL	29771946	148288	95081
	DUP	52583	133129	3650
	DEL	21013	1281	111866

Supplementary Table 3. Negative Predicted Value (NPV) score comparison of the WES-based CNV callers on the 1000 Genomes data set (test set). CNVnator calls on the matched WGS samples are used as the ground truth. Bold indicates the best result in that category.

**TOOLS**	**DEL NPV Score**	**DUP NPV Score**	**Overall NPV Score**
Control-FREEC	0.670	0.680	0.675
DECoNT-Control-FREEC	0.677	0.683	0.680
CNVkit	0.675	0.675	0.675
DECoNT-CNVkit	0.669	0.677	0.673
XHMM	0.994	0.991	0.992
DECoNT-XHMM	0.994	0.991	0.992
CONIFER	0.993	0.989	0.991
DECoNT-CONIFER	0.993	0.989	0.991
CODEX2	0.993	0.991	0.992
DECoNT-CODEX2	0.993	0.991	0.992
GATK	0.993	0.992	0.992
ECOLE	**0.997**	**0.995**	**0.996**

Supplementary Table 4. NPA (Specificity) score comparison of the WES-based CNV callers on the 1000 Genomes data set (test set). CNVnator calls on the matched WGS samples are used as the ground truth. Bold indicates the best result in that category

**TOOLS**	**DEL NPA Score**	**DUP NPA Score**	**Overall NPA Score**
Control-FREEC	0.538	0.92	0.729
DECoNT-Control-FREEC	0.398	0.968	0.683
CNVkit	0.694	0.912	0.803
DECoNT-CNVkit	0.836	0.838	0.837
XHMM	0.999	0.999	0.999
DECoNT-XHMM	**1.0**	**1.0**	**1.0**
CONIFER	**1.0**	0.999	**1.0**
DECoNT-CONIFER	**1.0**	**1.0**	**1.0**
CODEX2	0.818	0.843	0.831
DECoNT-CODEX2	0.941	0.94	0.941
GATK	0.962	0.954	0.958
ECOLE	0.999	0.998	0.999

Supplementary Table 5. Confusion Matrices for the performance comparison based on CNVnator calls used as the ground truth for the 1000 Genomes data set (28 samples obtained from S. Girirajan). These produce the precision and recall results in Table 1.

		**Ground Truth**		
TOOLS	**Predicted**	NO CALL	DUP	DEL
CNLearn	NO CALL	5324268	44745	38671
	DUP	1572	463	59
	DEL	883	6	81
ECOLE	NO CALL	5142291	24449	17584
	DUP	10369	23114	440
	DEL	3883	165	20160

Supplementary Table 6. Performance comparison on the NA12878 sample using various platforms. Bold indicates the best result in that category. CNVnator calls on the matched WGS sample are used as the semi-ground truth.

Platform	Tool	DEL Precision	DUP Precision	Overall Precision	DEL Recall	DUP Recall	Overall Recall	DEL F1 Score	DUP F1 Score	Overall F1 Score
BGI 500	Control-FREEC	0.150	0.285	0.218	0.009	0.338	0.174	0.017	0.309	0.193
	XHMM	0.042	0.006	0.024	0.010	0.001	0.006	0.016	0.002	0.010
	CONIFER	0.002	0.000	0.001	0.076	0.000	0.038	0.004	0.000	0.002
	CODEX2	0.007	0.007	0.007	0.179	0.101	0.140	0.013	0.013	0.013
	DECoNT-Control-FREEC	0.345	0.419	0.382	**0.665**	0.031	0.348	0.454	0.057	0.364
	DECoNT-XHMM	0.041	0.017	0.029	0.010	0.001	0.006	0.016	0.002	0.043
	DECoNT-CONIFER	0.010	0.001	0.006	0.052	0.010	0.031	0.017	0.002	0.010
	DECoNT-CODEX2	0.023	0.026	0.025	0.097	0.096	0.097	0.037	0.041	0.040
	ECOLE	**0.703**	**0.588**	**0.646**	0.473	**0.378**	**0.426**	**0.566**	**0.460**	**0.513**
HiSeq 4000	Control-FREEC	0.118	0.280	0.199	0.017	0.227	0.122	0.029	0.251	0.151
	XHMM	0.031	0.190	0.111	0.017	0.003	0.010	0.022	0.006	0.018
	CONIFER	0.035	0.000	0.018	0.019	0.000	0.010	0.025	0.000	0.013
	CODEX2	0.008	0.014	0.011	0.185	0.213	0.199	0.015	0.026	0.021
	DECoNT-Control-FREEC	0.337	0.426	0.382	0.455	0.024	0.240	0.387	0.045	0.294
	DECoNT-XHMM	0.043	0.017	0.030	0.016	0.001	0.009	0.023	0.002	0.014
	DECoNT-CONIFER	0.018	0.161	0.090	0.002	0.090	0.046	0.004	0.115	0.061
	DECoNT-CODEX2	0.007	0.007	0.007	0.034	0.075	0.055	0.012	0.013	0.012
	ECOLE	**0.926**	**0.514**	**0.720**	**0.538**	**0.450**	**0.494**	**0.680**	**0.480**	**0.586**
MGISEQ 2000	Control-FREEC	0.150	0.285	0.218	0.009	0.338	0.174	0.017	0.309	0.193
	XHMM	0.042	0.006	0.024	0.010	0.001	0.006	0.016	0.002	0.010
	CONIFER	0.002	0.000	0.001	0.076	0.000	0.038	0.004	0.000	0.002
	CODEX2	0.007	0.007	0.007	0.179	0.101	0.140	0.013	0.013	0.013
	DECoNT-Control-FREEC	0.345	0.419	0.382	**0.665**	0.031	0.348	0.454	0.057	0.364
	DECoNT-XHMM	0.041	0.017	0.029	0.010	0.001	0.006	0.016	0.002	0.043
	DECoNT-CONIFER	0.010	0.001	0.006	0.052	0.010	0.031	0.017	0.002	0.010
	DECoNT-CODEX2	0.023	0.026	0.025	0.097	0.096	0.097	0.037	0.041	0.040
	ECOLE	**0.689**	**0.587**	**0.638**	0.472	**0.381**	**0.427**	**0.560**	**0.462**	**0.512**
NovaSeq 6000	Control-FREEC	0.095	0.288	0.192	0.012	0.059	0.036	0.021	0.097	0.060
	XHMM	0.035	0.292	0.163	0.016	0.005	0.011	0.022	0.010	0.021
	CONIFER	0.000	0.000	0.000	0.000	0.000	0.000	0.000	0.000	0.000
	CODEX2	0.008	0.018	0.013	0.228	0.277	0.253	0.015	0.034	0.025
	DECoNT-Control-FREEC	0.278	0.406	0.342	0.106	0.016	0.061	0.153	0.031	0.104
	DECoNT-XHMM	0.049	0.095	0.072	0.015	0.003	0.009	0.023	0.006	0.016
	DECoNT-CONIFER	0.000	0.000	0.000	0.000	0.000	0.000	0.000	0.000	0.000
	DECoNT-CODEX2	0.016	0.015	0.016	0.118	0.181	0.150	0.028	0.028	0.029
	ECOLE	**0.710**	**0.542**	**0.626**	**0.361**	**0.468**	**0.415**	**0.479**	**0.502**	**0.499**

Supplementary Table 7. Confusion Matrices for the performance comparison based on CNVnator calls used as the ground truth for the NA12878 sample calls made using the BGISEQ-500 platform. These results partially produce the precision and recall results in Supplementary Table 6.

		**Ground Truth**		
TOOLS	**Predicted**	NO CALL	DUP	DEL
Control-FREEC	NO CALL	42188	37096	39088
	DUP	24112	20216	26429
	DEL	1098	2395	619
DECoNT-Control-FREEC	NO CALL	21936	18665	20979
	DUP	1408	1828	1121
	DEL	44054	39214	44036
XHMM	NO CALL	189471	1288	1763
	DUP	312	2	0
	DEL	328	60	17
DECoNT-XHMM	NO CALL	189670	1288	1763
	DUP	113	2	0
	DEL	328	60	17
CONIFER	NO CALL	125282	1106	1644
	DUP	0	0	0
	DEL	64829	244	136
DECoNT-CONIFER	NO CALL	168871	1165	1659
	DUP	12432	13	28
	DEL	8808	172	93
CODEX2	NO CALL	128842	960	1408
	DUP	18375	137	54
	DEL	42894	253	318
DECoNT-CODEX2	NO CALL	178118	1178	1573
	DUP	4771	129	34
	DEL	7222	43	173
ECOLE	NO CALL	189524	738	912
	DUP	332	511	26
	DEL	255	101	842

Supplementary Table 8. Confusion Matrices for the performance comparison based on CNVnator calls used as the ground truth for the NA12878 sample calls made using HiSeq 4000 platform. These results partially produce the precision and recall results in Supplementary Table 6.

		**Ground Truth**		
TOOLS	**Predicted**	NO CALL	DUP	DEL
Control-FREEC	NO CALL	48513	40206	46702
	DUP	16500	13543	18321
	DEL	2385	5958	1113
DECoNT-Control-FREEC	NO CALL	35959	29631	35210
	DUP	1096	1458	863
	DEL	30343	28618	30063
XHMM	NO CALL	189220	1304	1741
	DUP	8	4	9
	DEL	883	42	30
DECoNT-XHMM	NO CALL	189450	1314	1745
	DUP	51	1	6
	DEL	610	35	29
CONIFER	NO CALL	189323	1196	1746
	DUP	0	0	0
	DEL	788	154	34
DECoNT-CONIFER	NO CALL	189345	1220	1760
	DUP	613	121	17
	DEL	153	9	3
CODEX2	NO CALL	128405	759	1337
	DUP	20425	287	114
	DEL	41281	304	329
DECoNT-CODEX2	NO CALL	166852	1203	1574
	DUP	14341	101	145
	DEL	8918	46	61
ECOLE	NO CALL	189494	735	794
	DUP	548	608	28
	DEL	69	7	958

Supplementary Table 9. Confusion Matrices for the performance comparison based on CNVnator calls used as the ground truth for the NA12878 sample calls made using the MGISEQ-2000 platform. These results partially produce the precision and recall results in Supplementary Table 6.

		**Ground Truth**		
TOOLS	**Predicted**	NO CALL	DUP	DEL
Control-FREEC	NO CALL	30063	37096	39088
	DUP	24112	20216	26429
	DEL	1098	2395	619
DECoNT-Control-FREEC	NO CALL	21936	18665	20979
	DUP	1408	1828	1121
	DEL	44054	39214	44036
XHMM	NO CALL	189471	1288	1763
	DUP	312	2	0
	DEL	328	60	17
DECoNT-XHMM	NO CALL	189670	1288	1763
	DUP	113	2	0
	DEL	328	60	17
CONIFER	NO CALL	125282	1106	1644
	DUP	0	0	0
	DEL	64829	244	136
DECoNT-CONIFER	NO CALL	168871	1165	1659
	DUP	12432	13	28
	DEL	8808	172	93
CODEX2	NO CALL	128842	960	1408
	DUP	18375	137	54
	DEL	42894	253	318
DECoNT-CODEX2	NO CALL	178118	1178	1573
	DUP	4771	129	34
	DEL	7222	43	173
ECOLE	NO CALL	189497	735	913
	DUP	336	514	26
	DEL	278	101	841

Supplementary Table 10. Confusion Matrices for the performance comparison based on CNVnator calls used as the ground truth for the NA12878 sample calls made using NovaSeq 6000 platform. These results partially produce the precision and recall results in Supplementary Table 6.

		**Ground Truth**		
TOOLS	**Predicted**	NO CALL	DUP	DEL
Control-FREEC	NO CALL	61250	50528	60732
	DUP	4124	3540	4594
	DEL	2024	5639	810
DECoNT-Control-FREEC	NO CALL	58783	48391	58477
	DUP	761	952	631
	DEL	7854	10364	7028
XHMM	NO CALL	189382	1288	1743
	DUP	8	7	9
	DEL	721	55	28
DECoNT-XHMM	NO CALL	189595	1295	1753
	DUP	38	4	0
	DEL	478	51	27
CONIFER	NO CALL	190026	1346	1780
	DUP	0	0	0
	DEL	85	4	0
DECoNT-CONIFER	NO CALL	190026	1346	1780
	DUP	0	0	0
	DEL	85	4	0
CODEX2	NO CALL	122523	702	1161
	DUP	20298	374	213
	DEL	47290	274	406
DECoNT-CODEX2	NO CALL	161203	975	1375
	DUP	15728	245	195
	DEL	13180	130	210
ECOLE	NO CALL	189471	627	1073
	DUP	469	632	65
	DEL	171	91	642

Supplementary Table 11. The performance comparison of ECOLE on NA12878 samples sequenced with NimbleGen SeqCap v3 and SeqCap EZ Human Exome Library v3.0 capture kits

**Performance Metrics**	**Capture Kits**	
	SeqCap EZ Human Exome Library v3.0	NimbleGen SeqCap v3
DEL Precision	0.863	0.926
DUP Precision	0.545	0.514
Overall Precision	0.704	0.720
DEL Recall	0.510	0.538
DUP Recall	0.523	0.450
Overall Recall	0.517	0.494
DEL F1 Score	0.641	0.680
DUP F1 Score	0.534	0.480
Overall F1 Score	0.588	0.580

Supplementary Table 12. The performance comparison of ECOLE on exones whose read depths are captured with single and multiple probes using SeqCap EZ Human Exome Library v3.0 and NimbleGen SeqCap v3 capture kits.

Performance Metrics	Capture Kits			
	SeqCap EZ Human Exome Library v3.0		NimbleGen SeqCap v3	
	Single Probe Exons	Multiple Probe Exons	Single Probe Exons	Multiple Probe Exons
DEL Precision	0.875	0.845	0.927	0.500
DUP Precision	0.541	0.559	0.513	0.636
Overall Precision	0.708	0.702	0.720	0.568
DEL Recall	0.530	0.484	0.554	0.024
DUP Recall	0.588	0.524	0.538	0.135
Overall Recall	0.559	0.504	0.546	0.080
DEL F1 Score	0.660	0.616	0.694	0.045
DUP F1 Score	0.563	0.541	0.526	0.222
Overall F1 Score	0.612	0.579	0.610	0.134

Supplementary Table 13. The performance comparison of the WES-based CNV callers on the 1000 Genomes test set, which contains 157 samples. Chaisson et al.'s human expert-curated calls on the matched WGS samples are used as the ground truth [1]. We also used the DECoNT tool to polish call sets of all considered tools which are denoted by DECONT-*tool_name*. ECOLE$^{FT-EXPERT}$ corresponds to the fine-tuned version of ECOLE model. Bold indicates the best result in that category.

TOOLS	DEL Precision	DUP Precision	Overall Precision	DEL Recall	DUP Recall	Overall Recall	DEL F1 Score	DUP F1 Score	Overall F1 Score
XHMM	0.059	0.008	0.034	0.008	0.021	0.015	0.014	0.012	0.021
CONIFER	0.000	**1.000**	0.500	0.000	0.076	0.038	0.000	0.141	0.071
CODEX2	0.023	0.003	0.013	0.238	0.138	0.188	0.042	0.006	0.024
DECoNT-XHMM	0.051	0.010	0.031	0.006	0.019	0.013	0.011	0.013	0.018
DECoNT-CONIFER	0.000	**1.000**	0.500	0.000	0.061	0.031	0.000	0.115	0.058
DECoNT-CODEX2	0.022	0.004	0.013	0.070	0.087	0.079	0.033	0.008	0.022
ECOLE	0.181	0.070	0.126	0.050	0.137	0.094	0.078	0.093	0.108
ECOLE$^{FT-EXPERT}$	**0.584**	0.789	**0.687**	**0.444**	**0.547**	**0.496**	**0.504**	**0.646**	**0.576**

Supplementary Table 14. Confusion Matrices for the performance comparison based on Chaisson et al. calls used as the ground truth [1] for the 1000 Genomes data set (test set). These produce the precision and recall results in Supplementary Table 13.

		**Ground Truth**		
TOOLS	**Predicted**	NO CALL	DUP	DEL
XHMM	NO CALL	746801	2556	14310
	DUP	7149	56	67
	DEL	1894	12	119
DECoNT-XHMM	NO CALL	749417	2567	14342
	DUP	4877	51	70
	DEL	1550	6	84
CONIFER	NO CALL	188992	609	3590
	DUP	0	50	0
	DEL	0	0	0
DECoNT-CONIFER	NO CALL	188992	619	3590
	DUP	0	40	0
	DEL	0	0	0
CODEX2	NO CALL	481333	1704	8882
	DUP	130138	362	2161
	DEL	144373	558	3453
DECoNT-CODEX2	NO CALL	655677	2266	12338
	DUP	54791	228	1146
	DEL	45376	130	1012
ECOLE	NO CALL	748390	2224	13165
	DUP	4197	359	604
	DEL	3257	41	727
$ECOLE^{FT-EXPERT}$	NO CALL	751069	1136	7931
	DUP	254	1435	129
	DEL	4521	53	6436

Supplementary Table 15. Negative Predicted Value (NPV) scores comparison with other tools using Chaisson et. al labels as the ground truth [1]. Bold indicates the best result in that category.

**TOOLS**	**DEL NPV Score**	**DUP NPV Score**	**Overall NPV Score**
XHMM	0.981	0.997	0.989
DECoNT-XHMM	0.981	0.997	0.989
CONIFER	0.981	0.997	0.989
DECoNT-CONIFER	0.981	0.997	0.989
CODEX2	0.982	0.996	0.989
DECoNT-CODEX2	0.981	0.997	0.989
ECOLE	0.982	0.997	0.990
$ECOLE^{FT-EXPERT}$	**0.989**	**0.998**	**0.994**

Supplementary Table 16. NPA (Specificity) scores comparison with other tools using Chaisson et. al labels as the ground truth [1]. Bold indicates the best result in that category

**TOOLS**	**DEL NPA Score**	**DUP NPA Score**	**Overall NPA Score**
XHMM	0.997	0.991	0.994
DECoNT-XHMM	0.998	0.994	0.996
CONIFER	**1.0**	**1.0**	**1.0**
DECoNT-CONIFER	**1.0**	**1.0**	**1.0**
CODEX2	0.809	0.828	0.819
DECoNT-CODEX2	0.94	0.927	0.934
ECOLE	0.996	0.994	0.995
$ECOLE^{FT-EXPERT}$	0.994	**1.0**	0.997

Supplementary Table 17. Confusion Matrices for the performance comparison of calls, CNVnator calls used as the ground truth for the Somatic samples from Guo et. al. [4]. These results partially produce the precision and recall results in Table 3.

		**Ground Truth**		
TOOLS	**Predicted**	NO CALL	DUP	DEL
XHMM	NO CALL	1386707	951597	148878
	DUP	1147	27926	30
	DEL	5273	701	1886
DECoNT-XHMM	NO CALL	1387256	954512	148884
	DUP	1127	23602	178
	DEL	4744	2110	1732
ECOLE	NO CALL	1385260	968078	147230
	DUP	4561	10608	670
	DEL	3306	1538	2894
ECOLE$^{FT-SOMATIC}$	NO CALL	908337	585915	92415
	DUP	439066	372057	37171
	DEL	45724	22252	21208

Supplementary Table 18. The performance comparison of the WES-based CNV callers on the 1000 Genomes data set (test set) on CNV segments (merged exons). CNVnator calls on the matched WGS samples are used as the ground truth. CNVkit and Control-FREEC return exact (integer) copy number predictions, which are discretized into deletion, duplication, and no-call. We also used the DECoNT tool to polish call sets of all considered tools which are denoted by DECONT-*tool_name*. See Supplementary Table 19 for corresponding confusion matrices.

**TOOLS**	**CNV Segment Del Precision**	**CNV Segment Dup Precision**	**Overall Precision**
Control-FREEC	0.673	0.107	0.391
CNVkit	0.590	0.153	0.371
XHMM	0.434	0.455	0.445
CONIFER	0.404	0.205	0.305
CODEX2	0.191	0.044	0.118
DECoNT-Control-FREEC	0.661	0.418	0.540
DECoNT-CNVkit	0.604	0.584	0.594
DECoNT-XHMM	**0.757**	0.688	0.722
DECoNT-CONIFER	0.525	0.367	0.446
DECoNT-CODEX2	0.356	0.115	0.236
ECOLE	0.724	**0.721**	**0.723**

Supplementary Table 19. Confusion Matrices for the performance comparison of calls on CNV segments and CNVnator calls are grouped together (CNV segments) to be used as ground truth for the 1000 Genomes data set (test set). These produce the precision results in Supplementary Table 18.

		**Ground Truth**		
TOOLS	**Predicted**	NO CALL	DUP	DEL
Control-FREEC	NO CALL	1800	562	3585
	DUP	26154	2787	4986
	DEL	7962	2664	13788
DECoNT-Control-FREEC	NO CALL	0	0	0
	DUP	9770	1740	2783
	DEL	26146	4273	19576
CNVkit	NO CALL	83	157	1330
	DUP	2967	589	1791
	DEL	2589	421	2152
DECoNT-CNVkit	NO CALL	0	0	0
	DUP	66	35	122
	DEL	188	24	115
XHMM	NO CALL	0	0	0
	DUP	5428	5042	607
	DEL	4303	1473	4438
DECoNT-XHMM	NO CALL	6421	887	676
	DUP	2255	5338	161
	DEL	1055	290	4208
CONIFER	NO CALL	0	0	0
	DUP	292	111	137
	DEL	9	22	21
DECoNT-CONIFER	NO CALL	162	14	13
	DUP	89	76	42
	DEL	50	43	103
CODEX2	NO CALL	0	0	0
	DUP	119576	6655	24667
	DEL	95429	5868	23934
DECoNT-CODEX2	NO CALL	164350	2826	11550
	DUP	26112	4837	10943
	DEL	22606	2525	13909
ECOLE	NO CALL	9253	9003	21057
	DUP	5919	17124	719
	DEL	4352	250	11534

Supplementary Table 20. Performance comparison with CNLearn on 1000 Genome data when using CNV segments. The results of the 28 samples for which CNLearn calls are obtained via personal communication are shown. Bold indicates the best result in that category. See Supplementary Table 21 for corresponding confusion matrices.

**TOOLS**	**CNV Segment Del Precision**	**CNV Segment Dup Precision**	**Overall Precision**
CNLearn	0.171	0.246	0.209
ECOLE	**0.664**	**0.712**	**0.688**

Supplementary Table 21. Confusion Matrices for the performance comparison of calls on CNV segments and CNVnator calls are grouped together to be used as ground truth for the 1000 Genomes data set (28 samples obtained from S. Girirajan). These produce the precision and recall results in Supplementary Table 20.

		**Predicted**		
TOOLS	**Actual**	NO CALL	DUP	DEL
CNLearn	NO CALL	0	0	0
	DUP	92	35	22
	DEL	184	2	38
ECOLE	NO CALL	1371	1952	4054
	DUP	1033	2985	238
	DEL	892	89	1953

Supplementary Table 22. Performance comparison with other tools using Chaisson et. al labels as the ground truth [1]. Results for 4 samples from this study which are left out for testing are shown. This table considers grouped CNV calls (CNV segments). Bold indicates the best result in that category. See Supplementary Table 23 for corresponding confusion matrices.

**TOOLS**	**CNV Segment DEL Precision**	**CNV Segment DUP Precision**	**Overall Precision**
XHMM	0.225	0.054	0.140
CONIFER	0.000	**1.000**	0.500
CODEX2	0.409	0.019	0.214
DECoNT-XHMM	0.354	0.052	0.203
DECoNT-CONIFER	0.000	**1.000**	0.500
DECoNT-CODEX2	**0.664**	0.033	0.349
ECOLE	0.425	0.148	0.286
ECOLE$^{FT-EXPERT}$	0.487	0.799	**0.643**

Supplementary Table 23. Confusion Matrices for the performance comparison of calls on CNV segments and Chaisson et. al. calls used as the ground truth [1] for the 1000 Genomes data set (test set). These produce the precision results in Supplementary Table 22.

		**Ground Truth**		
TOOLS	**Predicted**	NO CALL	DUP	DEL
XHMM	NO CALL	0	0	0
	DUP	169	14	75
	DEL	108	9	34
DECoNT-XHMM	NO CALL	100	9	25
	DUP	128	10	55
	DEL	49	4	29
CONIFER	NO CALL	0	0	0
	DUP	0	6	0
	DEL	0	0	0
DECoNT-CONIFER	NO CALL	0	0	0
	DUP	1	2	0
	DEL	0	0	0
CODEX2	NO CALL	0	0	0
	DUP	1605	50	980
	DEL	1172	43	844
DECoNT-CODEX2	NO CALL	2263	51	597
	DUP	319	30	568
	DEL	188	11	393
ECOLE	NO CALL	347	55	634
	DUP	474	95	107
	DEL	194	9	143
ECOLE$^{FT-EXPERT}$	NO CALL	1245	50	2128
	DUP	57	300	28
	DEL	1532	11	1463

Supplementary Table 24. Performance comparison on the NA12878 sample on CNV segments (merged exons). CNVnator calls on the matched WGS samples are used as the ground truth. Bold indicates the best result in that category. See Supplementary Tables 25-28 for corresponding confusion matrices.

**Platform**	**Tool**	**CNV Segment Del Precision**	**CNV Segment Dup Precision**	**CNV Segment Overall Precision**
BGI 500	Control-FREEC	0.405	0.156	0.280
	XHMM	0.158	0.045	0.102
	CONIFER	0.051	0.000	0.026
	CODEX2	0.216	0.040	0.128
	DECoNT-Control-FREEC	0.537	0.194	0.366
	DECoNT-XHMM	0.176	0.076	0.126
	DECoNT-CONIFER	0.096	0.010	0.053
	DECoNT-CODEX2	0.436	0.133	0.285
	ECOLE	**0.625**	**0.713**	**0.669**
HiSeq 4000	Control-FREEC	0.328	0.171	0.250
	XHMM	0.095	0.500	0.298
	CONIFER	0.173	0.000	0.087
	CODEX2	0.180	0.030	0.105
	DECoNT-Control-FREEC	0.418	0.180	0.300
	DECoNT-XHMM	0.158	0.000	0.079
	DECoNT-CONIFER	0.125	0.500	0.313
	DECoNT-CODEX2	0.331	0.067	0.200
	ECOLE	**0.810**	**0.508**	**0.659**
MGISEQ 2000	Control-FREEC	0.405	0.156	0.280
	XHMM	0.158	0.045	0.102
	CONIFER	0.051	0.000	0.026
	CODEX2	0.216	0.040	0.128
	DECoNT-Control-FREEC	0.537	0.194	0.366
	DECoNT-XHMM	0.176	0.076	0.126
	DECoNT-CONIFER	0.096	0.010	0.053
	DECoNT-CODEX2	0.436	0.133	0.285
	ECOLE	**0.615**	**0.682**	**0.648**
NovaSeq 6000	Control-FREEC	0.333	0.195	0.264
	XHMM	0.081	**0.666**	0.374
	CONIFER	0.000	0.000	0.000
	CODEX2	0.189	0.043	0.116
	DECoNT-Control-FREEC	0.362	0.226	0.294
	DECoNT-XHMM	0.115	0.200	0.158
	DECoNT-CONIFER	0.000	0.000	0.000
	DECoNT-CODEX2	0.308	0.135	0.222
	ECOLE	**0.708**	0.593	**0.651**

Supplementary Table 25. Confusion Matrices for the performance comparison of calls on CNV segments and CNVnator calls used as the ground truth for the NA12878 sample calls made using the BGISEQ-500 platform. These results partially produce the precision results in Supplementary Table 24.

		**Ground Truth**		
TOOLS	**Predicted**	NO CALL	DUP	DEL
Control-FREEC	NO CALL	16	9	71
	DUP	49	23	80
	DEL	50	19	50
DECoNT-Control-FREEC	NO CALL	0	0	0
	DUP	9	6	19
	DEL	106	45	182
XHMM	NO CALL	0	0	0
	DUP	21	1	0
	DEL	10	6	3
DECoNT-XHMM	NO CALL	10	1	0
	DUP	12	1	0
	DEL	9	5	3
CONIFER	NO CALL	0	0	0
	DUP	0	0	0
	DEL	5301	58	308
DECoNT-CONIFER	NO CALL	7076	48	280
	DUP	2022	24	152
	DEL	1504	44	184
CODEX2	NO CALL	0	0	0
	DUP	640	32	136
	DEL	500	22	148
DECoNT-CODEX2	NO CALL	478	9	35
	DUP	31	8	24
	DEL	55	3	45
ECOLE	NO CALL	48	30	114
	DUP	20	62	6
	DEL	36	0	60

Supplementary Table 26. Confusion Matrices for the performance comparison of calls on CNV segments and CN-Vnator calls used as the ground truth for the NA12878 sample calls made using HiSeq 4000 platform. These results partially produce the precision results in Supplementary Table 24.

		**Ground Truth**		
TOOLS	**Predicted**	NO CALL	DUP	DEL
Control-FREEC	NO CALL	22	6	49
	DUP	27	14	44
	DEL	125	53	97
DECoNT-Control-FREEC	NO CALL	0	0	0
	DUP	17	7	15
	DEL	157	66	175
XHMM	NO CALL	0	0	0
	DUP	0	1	1
	DEL	79	16	12
DECoNT-XHMM	NO CALL	27	10	0
	DUP	6	0	2
	DEL	46	7	11
CONIFER	NO CALL	0	0	0
	DUP	0	0	0
	DEL	30	13	10
DECoNT-CONIFER	NO CALL	34	12	10
	DUP	4	8	6
	DEL	22	6	4
CODEX2	NO CALL	0	0	0
	DUP	1006	38	214
	DEL	754	38	184
DECoNT-CODEX2	NO CALL	607	9	54
	DUP	163	15	49
	DEL	104	7	58
ECOLE	NO CALL	76	45	151
	DUP	82	91	9
	DEL	15	1	68

Supplementary Table 27. Confusion Matrices for the performance comparison of calls on CNV segments and CNVnator calls used as the ground truth for the NA12878 sample calls made using the MGISEQ-2000 platform. These results partially produce the precision results in Supplementary Table 24.

		**Ground Truth**		
TOOLS	**Predicted**	NO CALL	DUP	DEL
Control-FREEC	NO CALL	16	9	71
	DUP	49	23	80
	DEL	50	19	50
DECoNT-Control-FREEC	NO CALL	0	0	0
	DUP	9	6	19
	DEL	106	45	182
XHMM	NO CALL	0	0	0
	DUP	21	1	0
	DEL	10	6	3
DECoNT-XHMM	NO CALL	10	1	0
	DUP	12	1	0
	DEL	9	5	3
CONIFER	NO CALL	0	0	0
	DUP	0	0	0
	DEL	5301	58	308
DECoNT-CONIFER	NO CALL	7076	48	280
	DUP	2022	24	152
	DEL	1504	44	184
CODEX2	NO CALL	0	0	0
	DUP	640	32	136
	DEL	500	22	148
DECoNT-CODEX2	NO CALL	478	9	35
	DUP	31	8	24
	DEL	55	3	45
ECOLE	NO CALL	53	27	113
	DUP	23	60	6
	DEL	37	0	59

Supplementary Table 28. Confusion Matrices for the performance comparison of calls on CNV segments and CNVnator calls used as the ground truth for the NA12878 sample calls made using NovaSeq 6000 platform. These results partially produce the precision results in Supplementary Table 24.

		**Ground Truth**		
TOOLS	**Predicted**	NO CALL	DUP	DEL
Control-FREEC	NO CALL	15	4	26
	DUP	19	8	19
	DEL	103	49	86
DECoNT-Control-FREEC	NO CALL	0	0	0
	DUP	11	7	15
	DEL	126	54	116
XHMM	NO CALL	0	0	0
	DUP	0	2	1
	DEL	65	14	10
DECoNT-XHMM	NO CALL	24	6	2
	DUP	4	1	1
	DEL	37	9	8
CONIFER	NO CALL	0	0	0
	DUP	0	0	0
	DEL	8	2	0
DECoNT-CONIFER	NO CALL	0	0	0
	DUP	0	0	0
	DEL	16	4	0
CODEX2	NO CALL	0	0	0
	DUP	1226	64	196
	DEL	884	66	236
DECoNT-CODEX2	NO CALL	840	14	43
	DUP	104	26	68
	DEL	107	12	57
ECOLE	NO CALL	60	42	141
	DUP	50	83	8
	DEL	27	1	68

Supplementary Table 29. Performance comparison with the SVM baseline model using the test set from 1000 Genome data. We use the exon-level semi-ground truth labels obtained using CNVnator. Bold indicates the best result in that category.

**TOOLS**	**DEL Precision**	**DEL Recall**	**DUP Precision**	**DUP Recall**	**Overall Precision**	**Overall Recall**
SVM	0.042	0.627	0.017	**0.543**	0.029	0.585
XGBoost	0.728	0.0208	0.561	0.0186	0.644	0.0197
ECOLE	**0.834**	**0.531**	**0.703**	0.470	**0.769**	**0.501**

Supplementary Table 30. The performance comparison of ECOLE on chromosome 21 and 10, against convolutional neural network (CNN)-based models that are trained and tested on chromosome 21 and 10.

**Model**	**DEL Precision**	**DUP Precision**	**Overall Precision**	**DEL Recall**	**DUP Recall**	**Overall Recall**	**DEL F1 Score**	**DUP F1 Score**	**Overall F1 Score**
Chromosome-Specific CNN (chr 21)	0.466	0.711	0.588	0.0376	0.00144	0.0195	0.0696	0.00287	0.0362
ECOLE (only chr 21)	0.760	0.950	0.855	0.959	0.893	0.926	0.848	0.920	0.884
Chromosome-Specific CNN (chr 10)	0.0	0.0	0.0	0.0	0.0	0.0	0.0	0.0	0.0
ECOLE (only chr 10)	0.0	0.0753	0.0377	0.0	0.585	0.292	0.0	0.133	0.0667

Supplementary Table 31. Performance comparison with Base Transformer using the test set from 1000 Genome data. We use the exon-level semi-ground truth labels obtained using CNVnator. Bold indicates the best result in that category.

TOOLS	DEL Precision	DEL Recall	DUP Precision	DUP Recall	Overall Precision	Overall Recall
Transformer base	0.625	0.00007	0.312	0.007	0.469	0.004
ECOLE	**0.834**	**0.531**	**0.703**	**0.470**	**0.769**	**0.501**

Supplementary Table 32. The performance comparison of ECOLE models trained with different sized inputs (100, 200, 500 and 1000). The performance is shown for calls shorter than 100, calls between 100 and 200, and calls larger than 200.

Call sizes	ECOLE input size	DEL Precision	DEL Recall	DUP Precision	DUP Recall	Overall Precision	Overall Recall	DEL F1 Score	DUP F1 Score	Overall F1 Score
For calls <100	100	0.786	**0.525**	0.0	0.0	0.393	**0.262**	**0.629**	0.0	**0.314**
	200	**0.987**	0.268	0.0	0.0	**0.493**	0.134	0.421	0.0	0.210
	500	0.994	0.212	0.0	0.0	0.492	0.106	0.349	0.0	0.176
	1000	0.533	0.166	0.0	0.0	0.266	0.083	0.253	0.0	0.126
For calls 100-200	100	0.0	0.0	0.0	0.0	0.0	0.0	0.0	0.0	0.0
	200	0.783	0.486	**0.755**	0.352	0.769	0.419	0.599	0.480	0.540
	500	**0.846**	0.298	0.724	0.378	**0.785**	0.338	0.440	0.496	0.468
	1000	0.533	**0.704**	0.590	**0.660**	0.562	**0.682**	**0.606**	**0.623**	**0.615**
For calls >200	100	0.0	0.0	0.0	0.0	0.0	0.0	0.0	0.0	0.0
	200	0.0	0.0	0.0	0.0	0.0	0.0	0.0	0.0	0.0
	500	**0.865**	0.349	**0.710**	0.352	**0.794**	0.363	0.497	0.470	0.483
	1000	0.620	**0.788**	0.535	**0.641**	0.578	**0.715**	**0.694**	**0.583**	**0.639**

2 Supplementary Figures

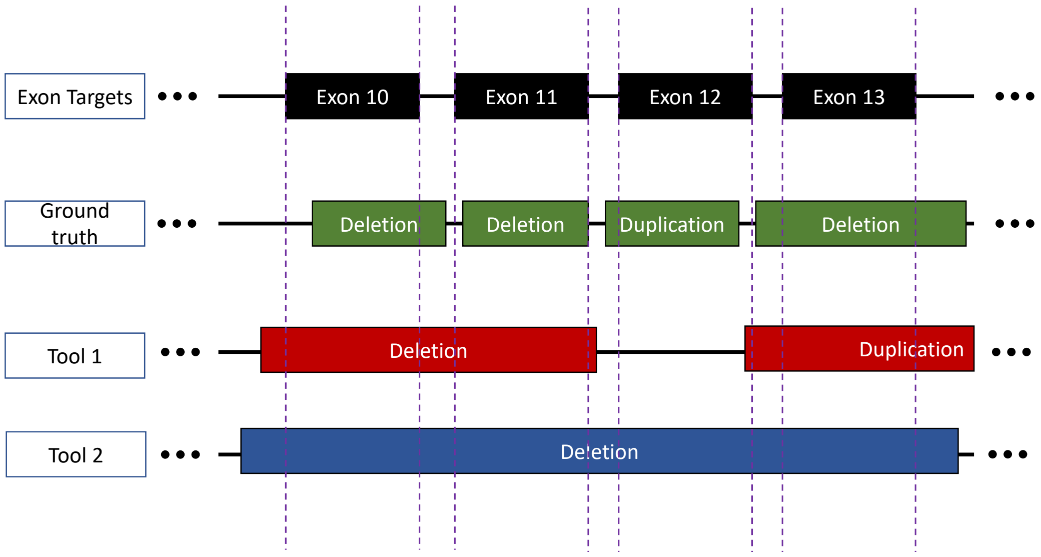

Supplementary Figure 1. The ground truths of exon-level CNV calling, introduced in Section 2.2, are obtained by intersecting the CNV call segments with the exons. Moreover, the CNV calls made by the tools are also obtained by intersecting them with the exons, thus we have fair comparison between different tools. If the tool or the ground truth did not report a CNV call in the target exon, then there is a no-call.In this case, we have a unique ground truth. In this example, Tool 1 has 2 TP calls for exons 10 and 11. It has 1 FP call for exon 13. Tool 2 has 3 TP calls for exons 10, 11, and 13. It has 1 FP call for exon 12. See Supplementary Figure 11 for the alternative approach.

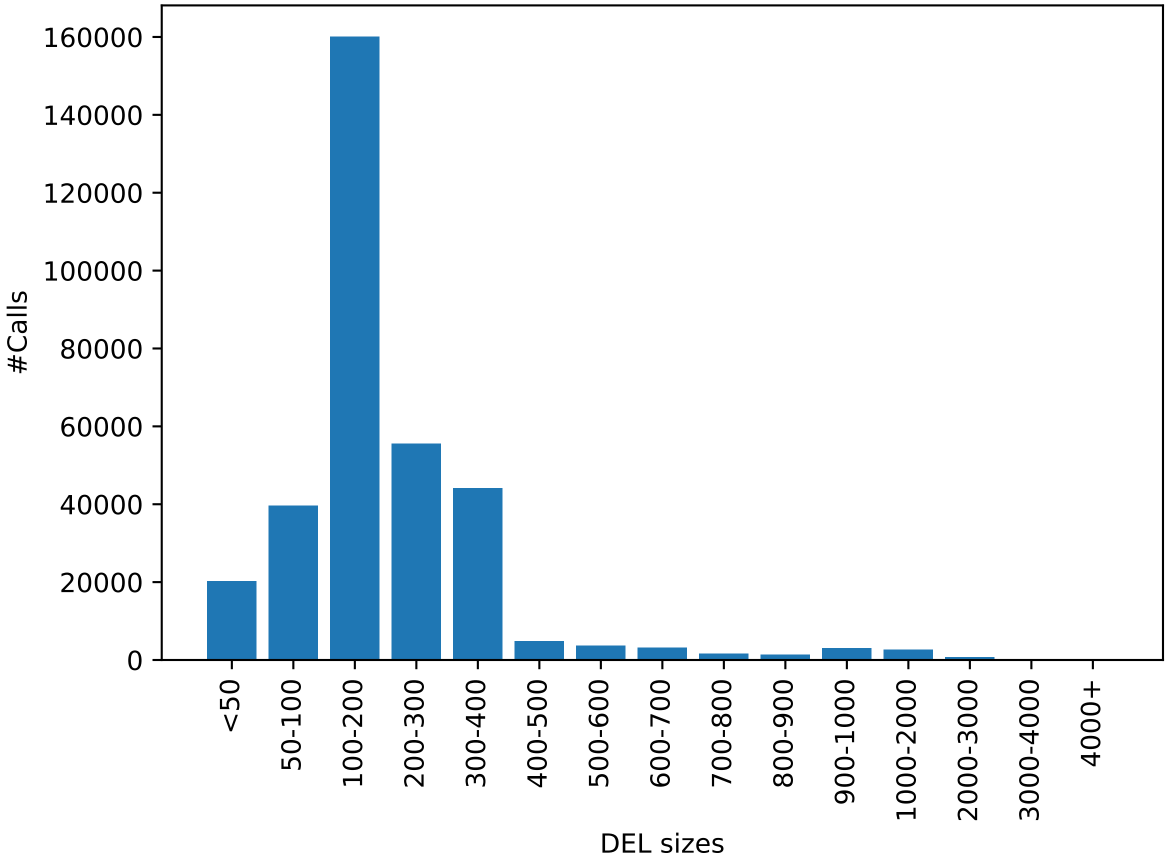

Supplementary Figure 2. Distribution of deletion call sizes in CNVnator predictions on the (WGS) 1000 Genomes data set (training set). Source data are provided as a Source Data file.

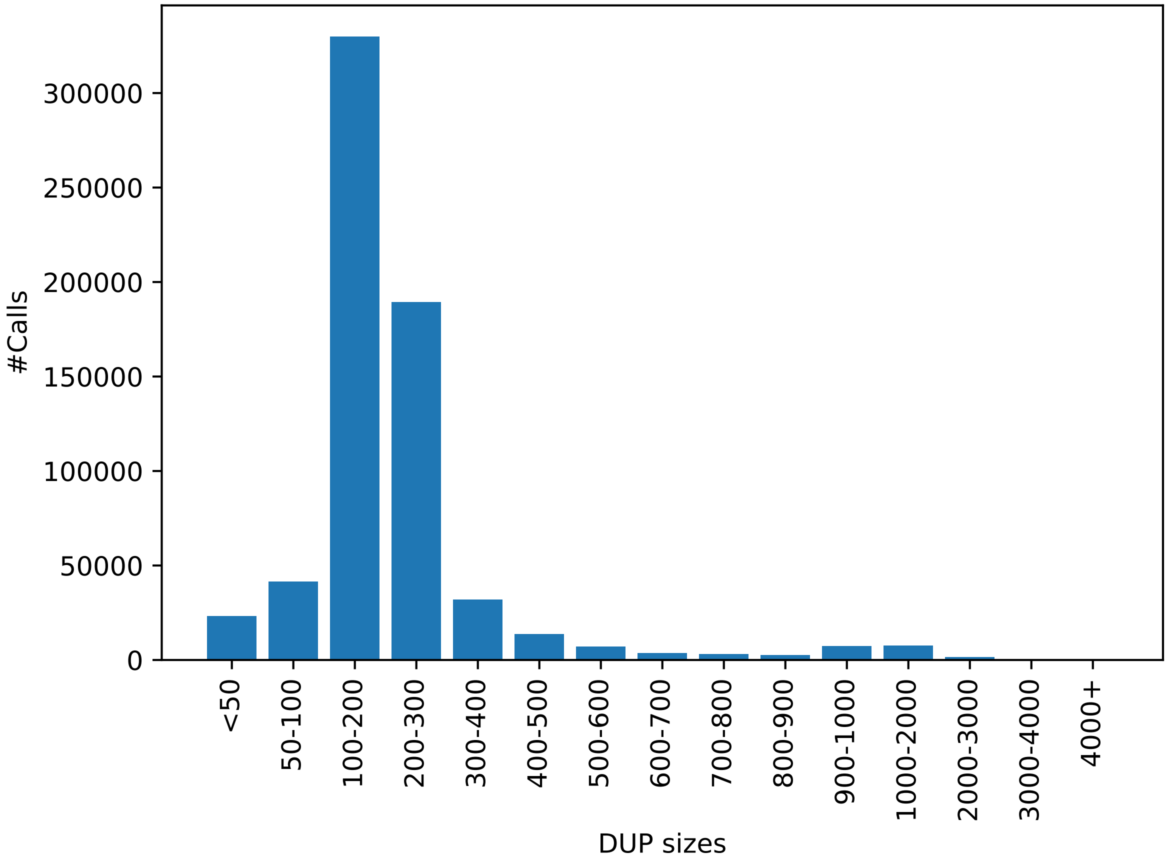

Supplementary Figure 3. Distribution of duplication call sizes in CNVnator predictions on the (WGS) 1000 Genomes data set (training set). Source data are provided as a Source Data file.

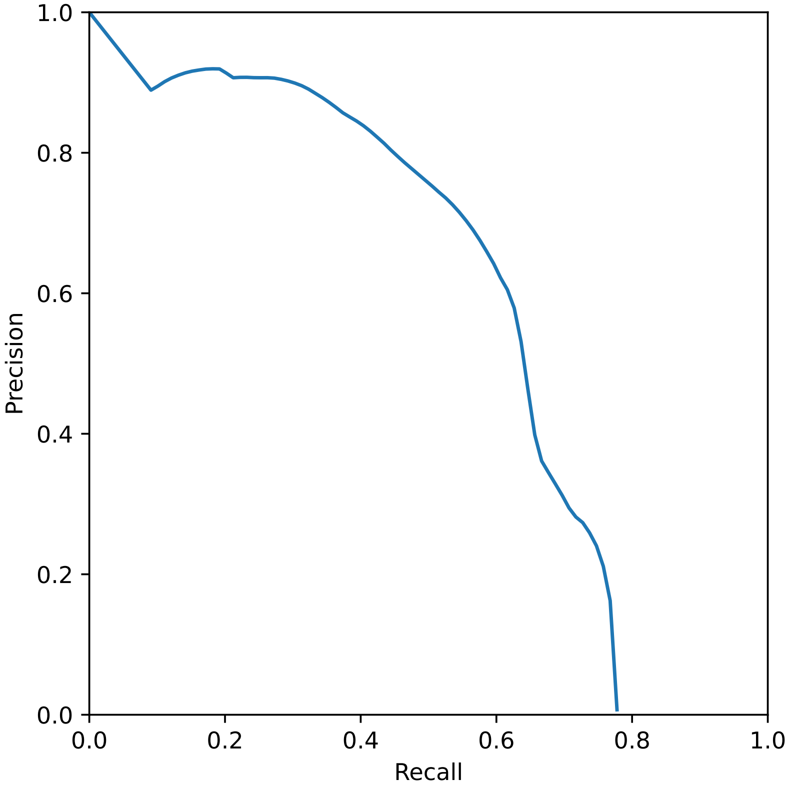

Supplementary Figure 4. The macro average precision-recall curve of ECOLE on 1000 Genomes data set (test set). The AUPR score of the model is 0.579. Source data are provided as a Source Data file.

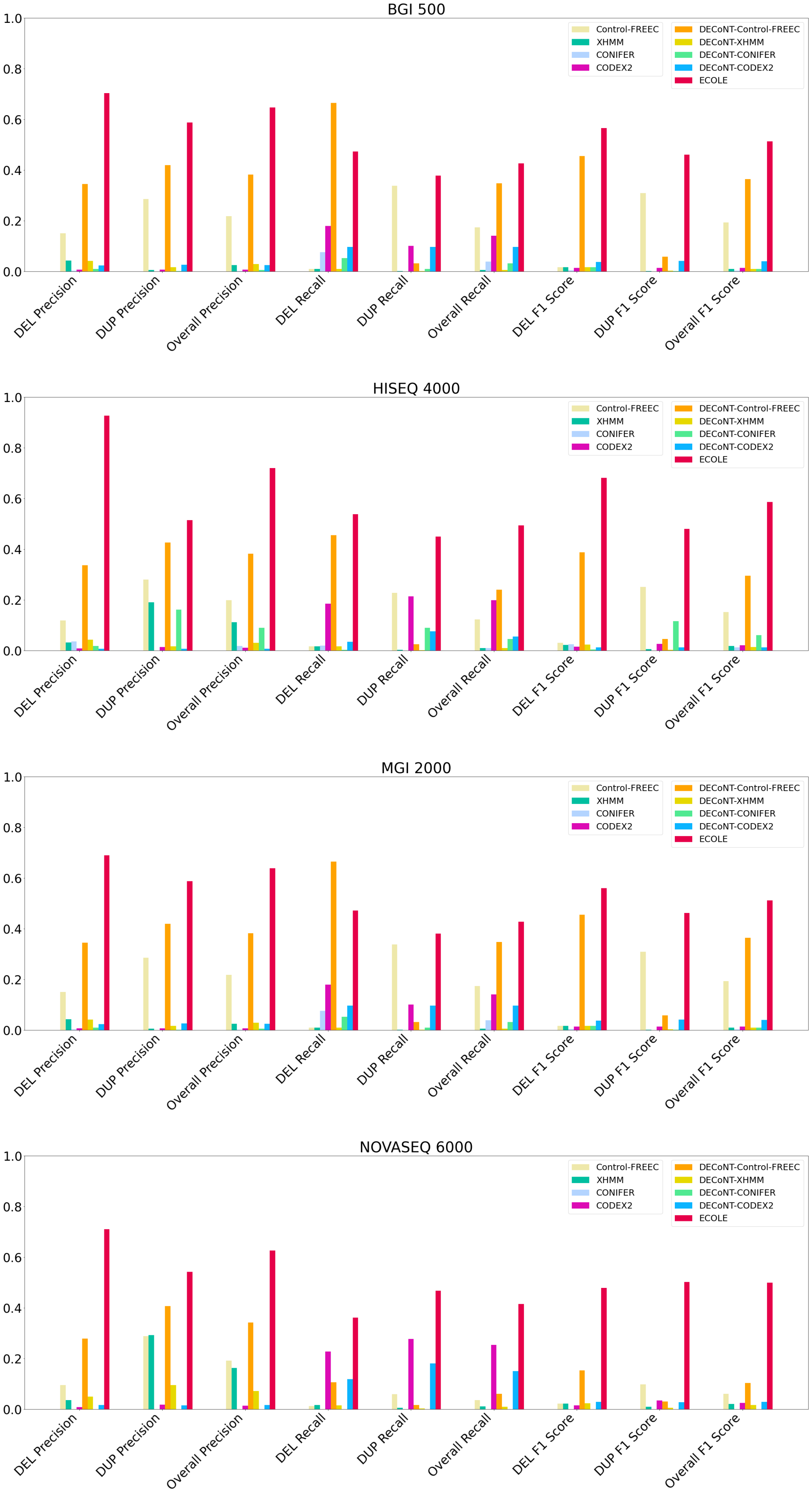

Supplementary Figure 5. Performance comparison with other tools on NA12878 sample. See Supplementary Table 6 for the values used to produce this plot and see Supplementary Tables 7-10 for the corresponding confusion matrices. Source data are provided as a Source Data file.

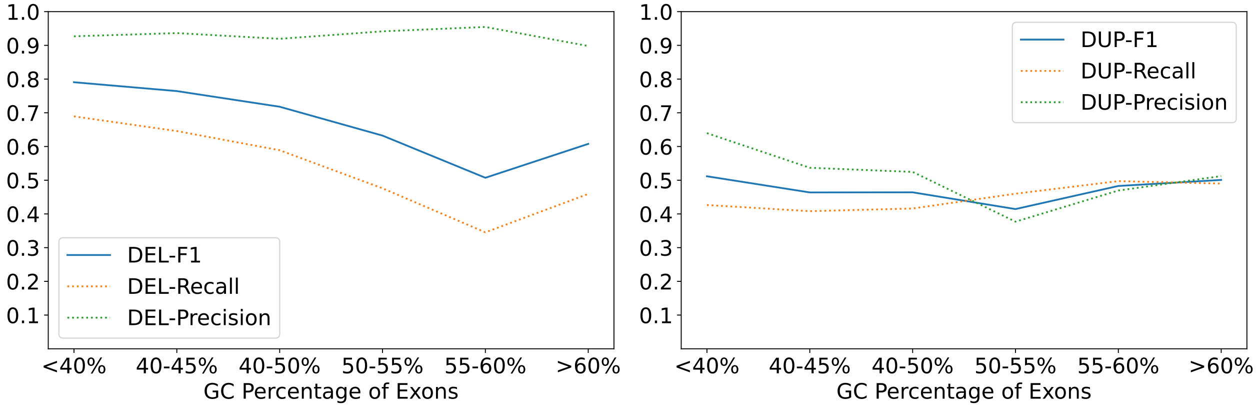

Supplementary Figure 6. The performance of ECOLE on exons with different GC content rates for NA12878 sample sequenced with NimbleGen SeqCap v3 capture kit. Source data are provided as a Source Data file.

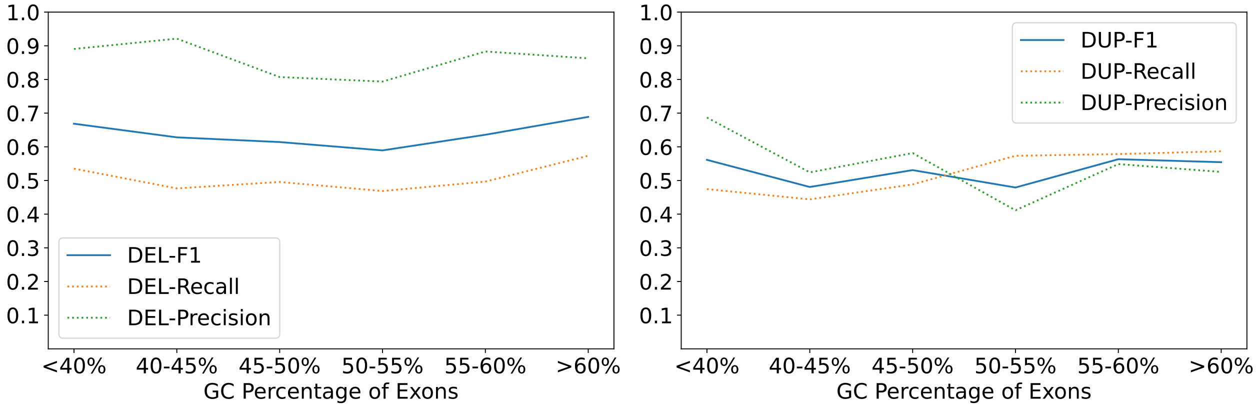

Supplementary Figure 7. The performance of ECOLE on exons with different GC content rates for NA12878 sample sequenced with SeqCap EZ Human Exome Library v3.0 capture kit. Source data are provided as a Source Data file.

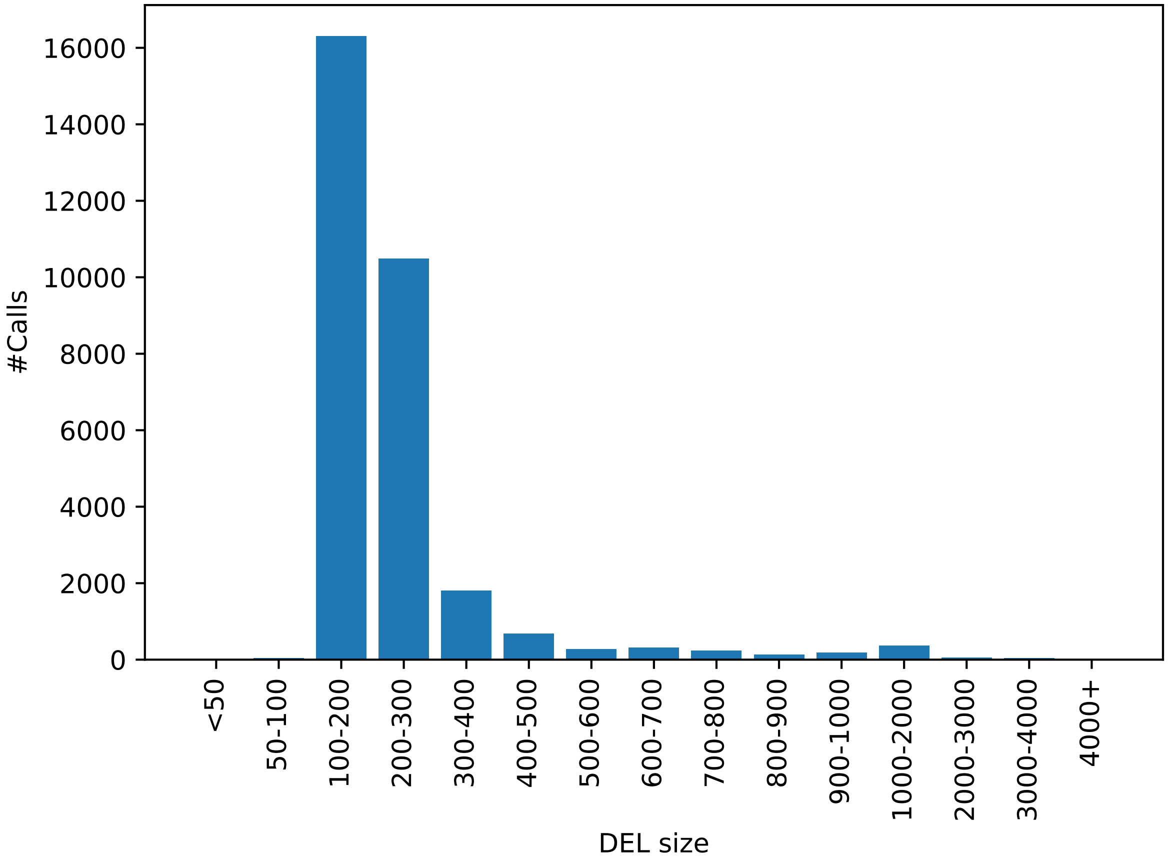

Supplementary Figure 8. Distribution of deletion ground-truth call sizes in Chaisson et. al. data set (test set). Source data are provided as a Source Data file.

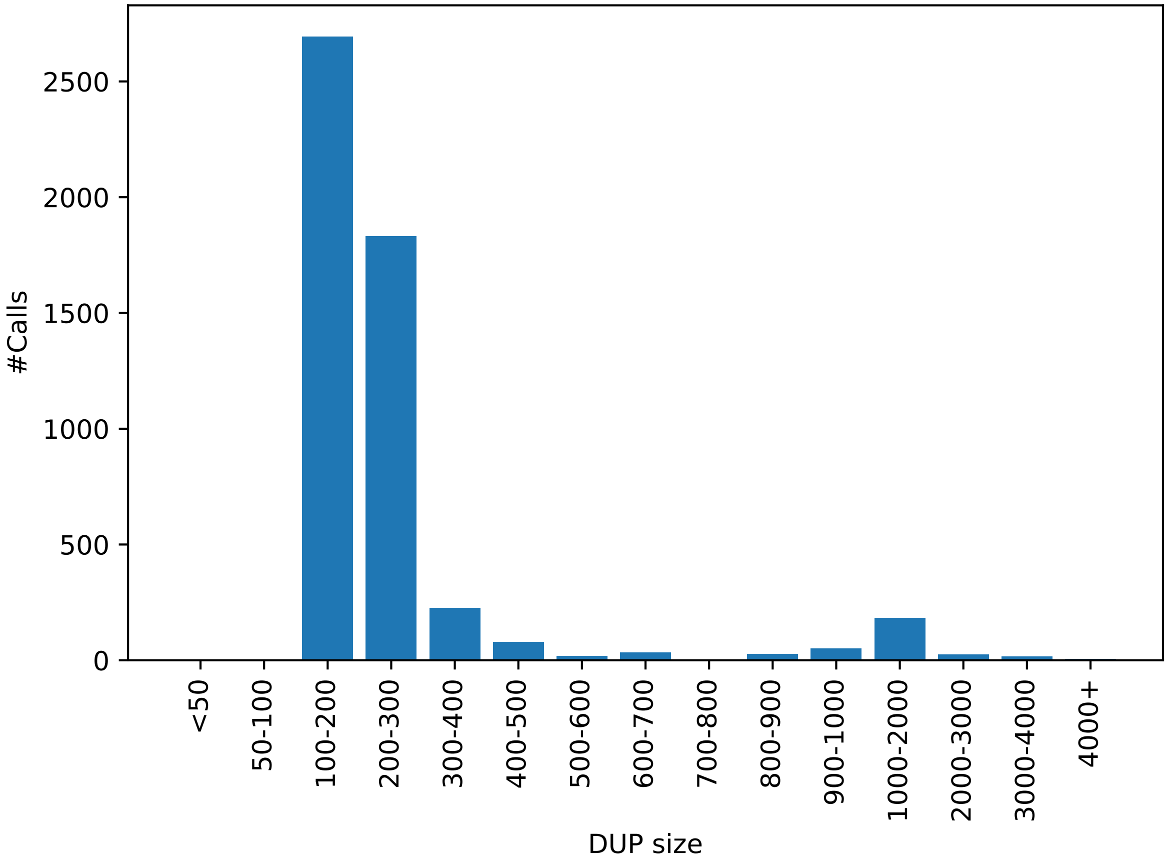

Supplementary Figure 9. Distribution of duplication ground-truth call sizes in Chaisson et. al. data set (test set). Source data are provided as a Source Data file.

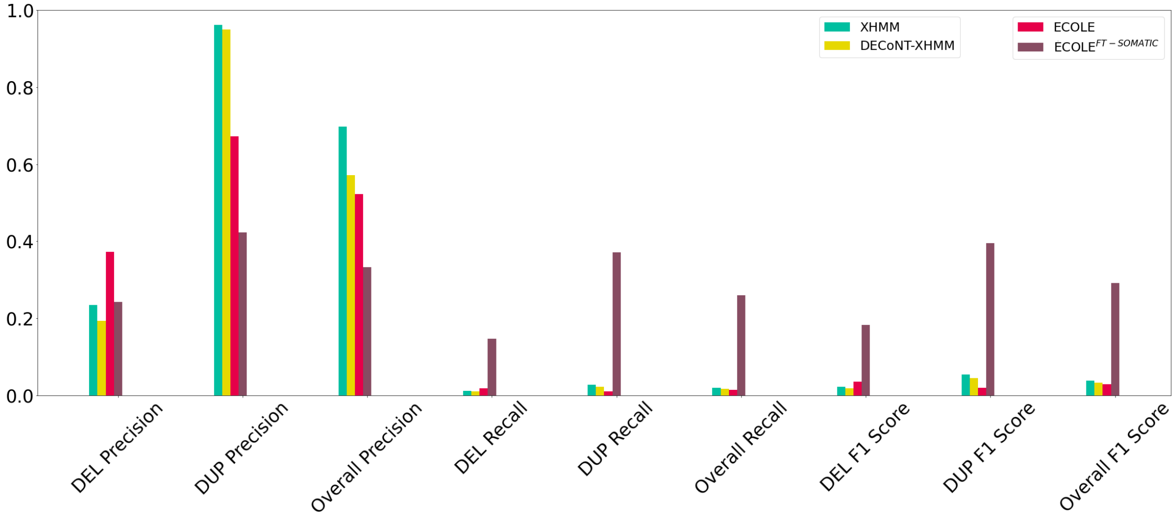

Supplementary Figure 10. The performance comparison with other tools on samples from Guo et. al. [4]. See Table 3 for the values used to produce this plot, and see Supplementary Table 17 for the corresponding confusion matrix. Source data are provided as a Source Data file.

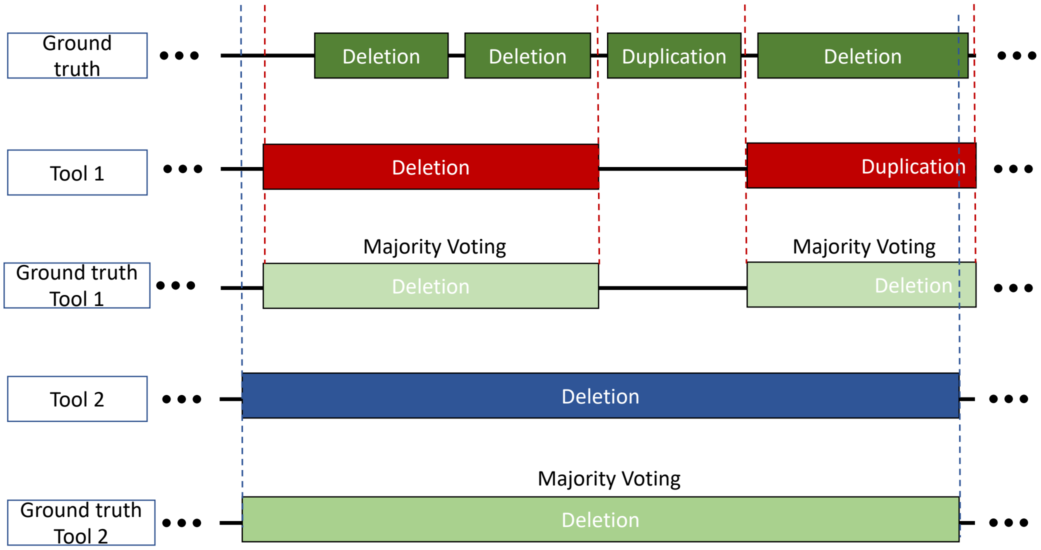

Supplementary Figure 11. The ground truths of merged CNV segment comparison, introduced in Section 2.3, are obtained by applying majority voting on the ground truth calls inside the target region of a CNV call made by a tool. Note that the ground truth labels are always shorter in length so majority voting is necessary to obtain the respective ground truth for a merged CNV call made by a tool. In this case, we have multiple ground truths for each tool, which prohibit the calculation of the recall fairly. Here, Tool 1 has 1 TP deletion call as the first two exons are grouped w.r.t. the calls of Tool 1, and a consensus ground truth is assigned. It has a single FP call for the last exon. Tool 2 has 1 TP deletion call as all exons are grouped due to the large call of Tool 2 which spans all exons. The consensus is that these exons have a deletion label w.r.t. majority voting.

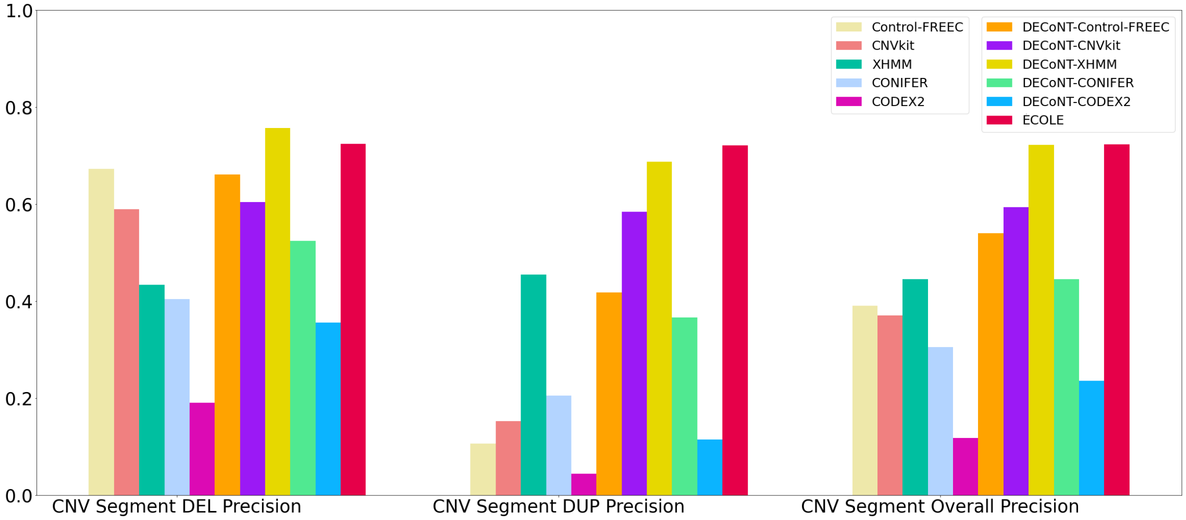

Supplementary Figure 12. The performance comparison of the WES-based CNV callers on the 1000 Genomes data set (test set) on CNV segments. CNVnator calls on the matched WGS samples are used as the ground truth. CNVkit and Control-FREEC return exact (integer) copy number predictions, which are discretized into deletion, duplication, and no-call. We also used the DECoNT tool to polish call sets of all considered tools which are denoted by DECONT-*tool_name*. Source data are provided as a Source Data file.

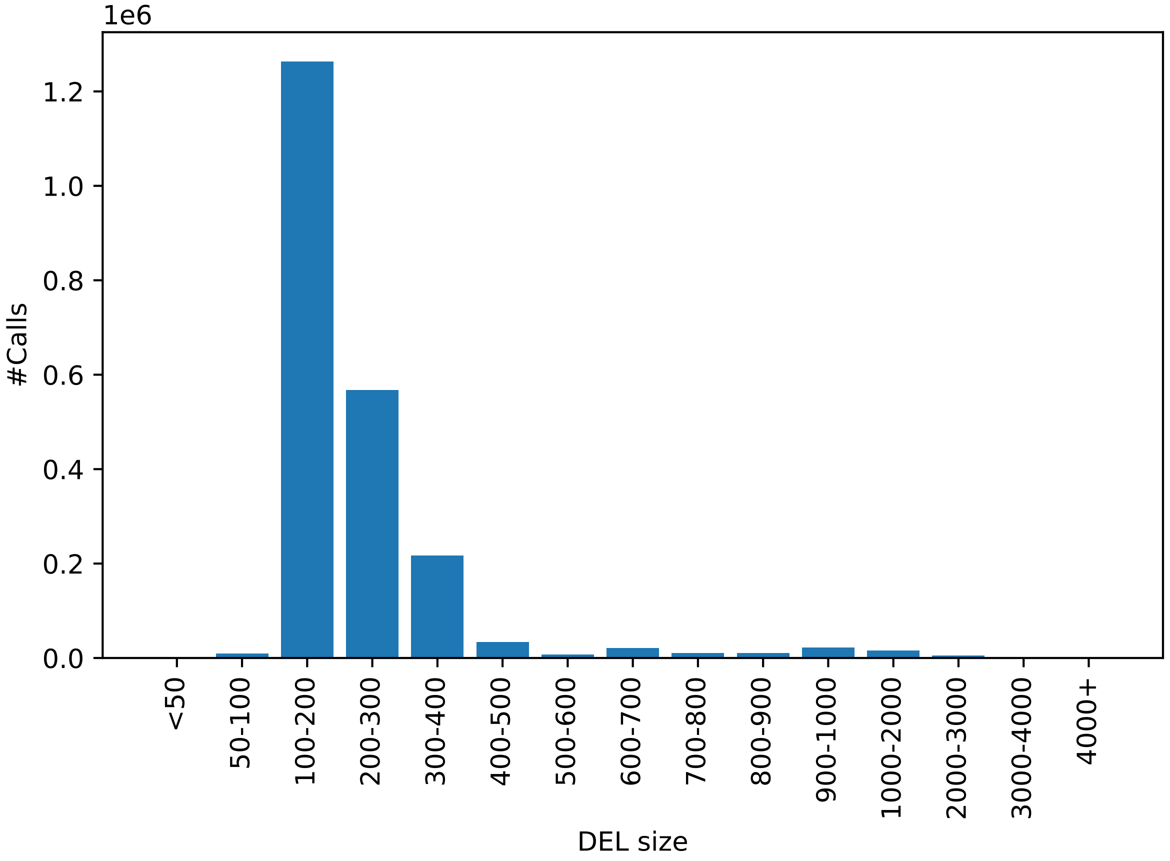

Supplementary Figure 13. Distribution of deletion call (merged CNV segments) sizes in CNVnator predictions on the (WGS) 1000 Genomes data set (training set). Source data are provided as a Source Data file.

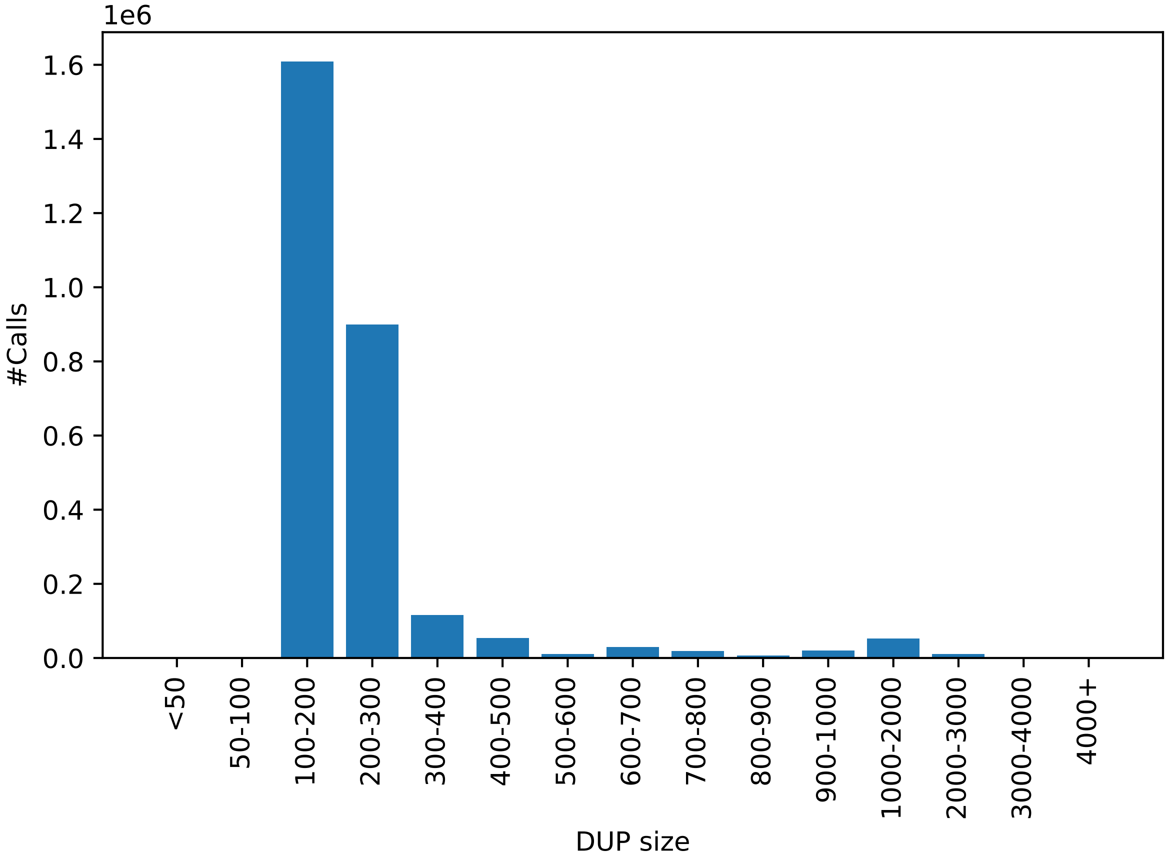

Supplementary Figure 14. Distribution of duplication call (merged CNV segments) sizes in CNVnator predictions on the (WGS) 1000 Genomes data set (training set). Source data are provided as a Source Data file.

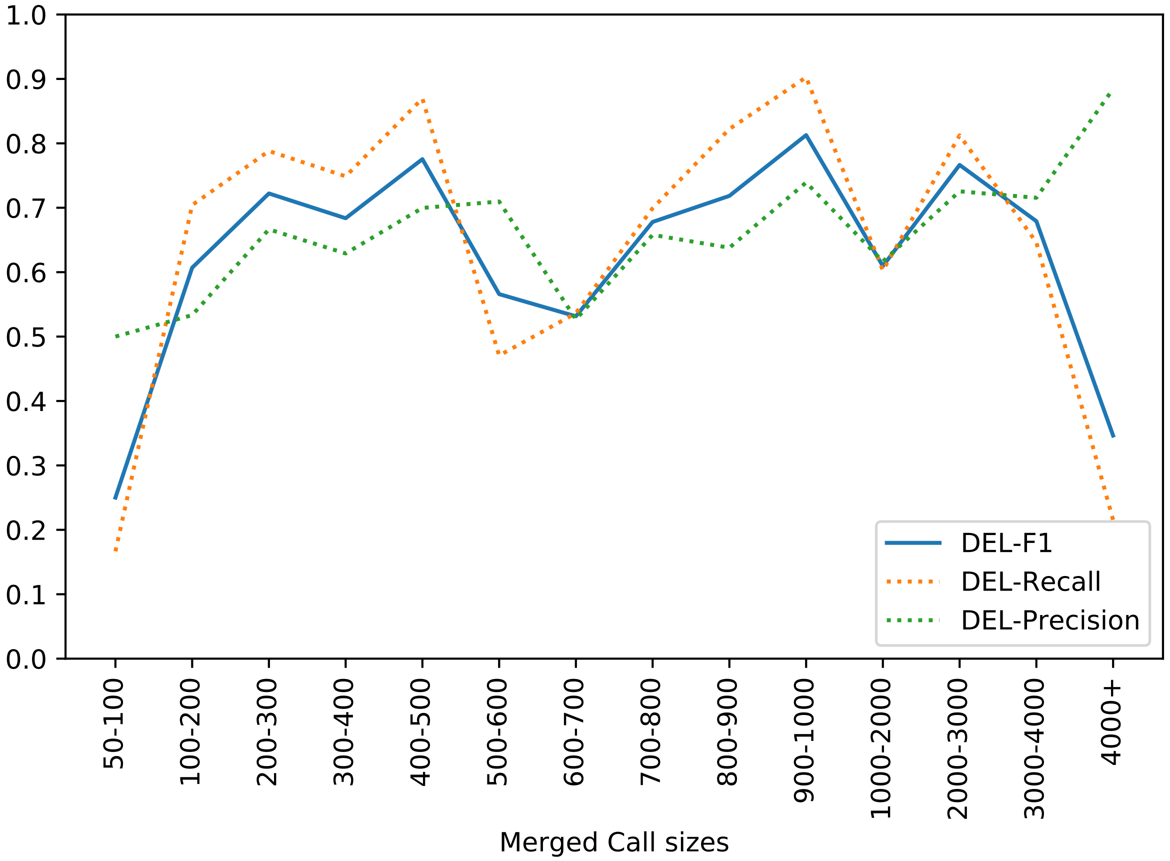

Supplementary Figure 15. Deletion detection performance (F1 score, Recall, Precision) of ECOLE across different call sizes for merged calls. Source data are provided as a Source Data file.

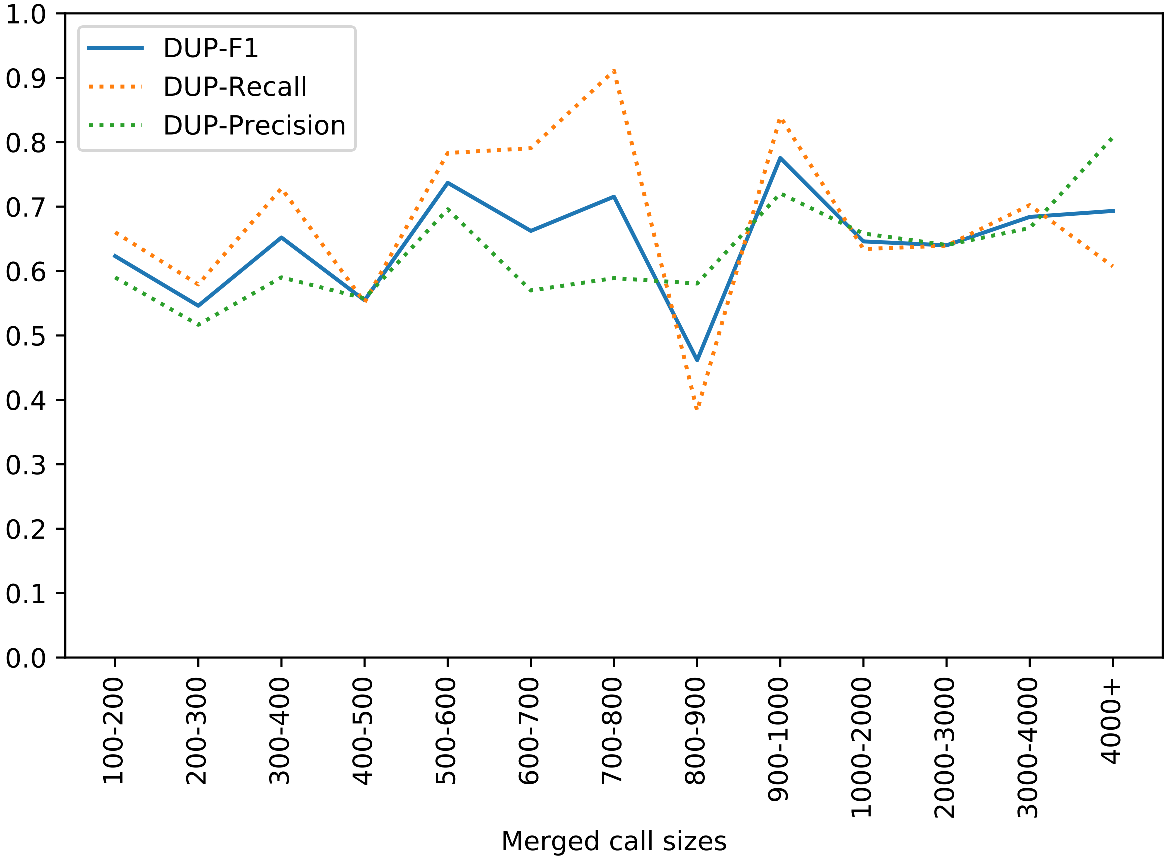

Supplementary Figure 16. Duplication detection performance (F1 score, Recall, Precision) of ECOLE across different call sizes for merged calls. Source data are provided as a Source Data file.

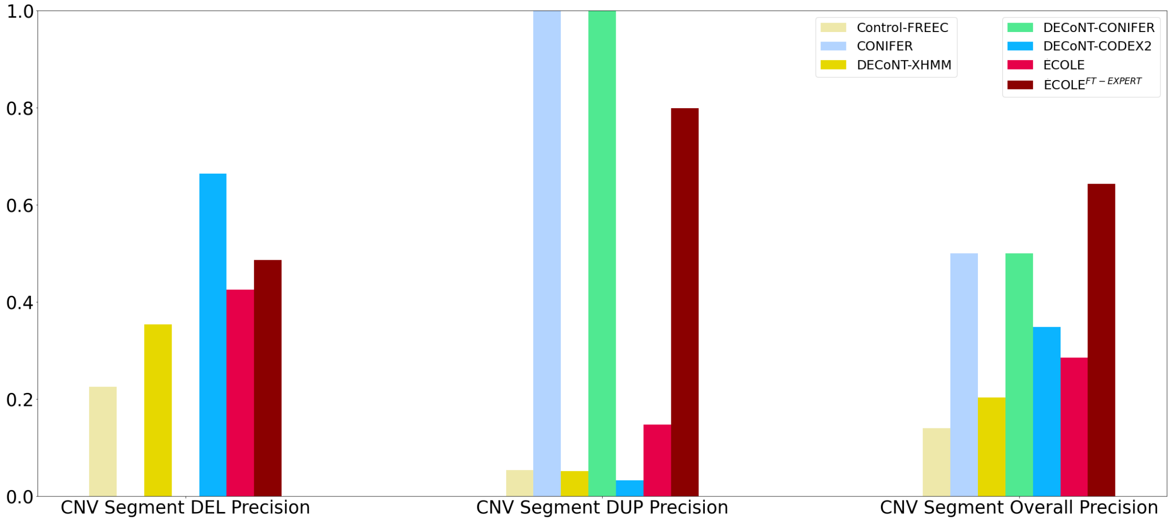

Supplementary Figure 17. The performance comparison of the WES-based CNV callers on the Chaisson et. al. data set (test set) on CNV segments. CNVnator calls on the matched WGS samples are used as the ground truth. Control-FREEC returns exact (integer) copy number predictions, which are discretized into deletion, duplication, and no-call. We also used the DECoNT tool to polish call sets of all considered tools which are denoted by DECONT-*tool_name*. Source data are provided as a Source Data file.

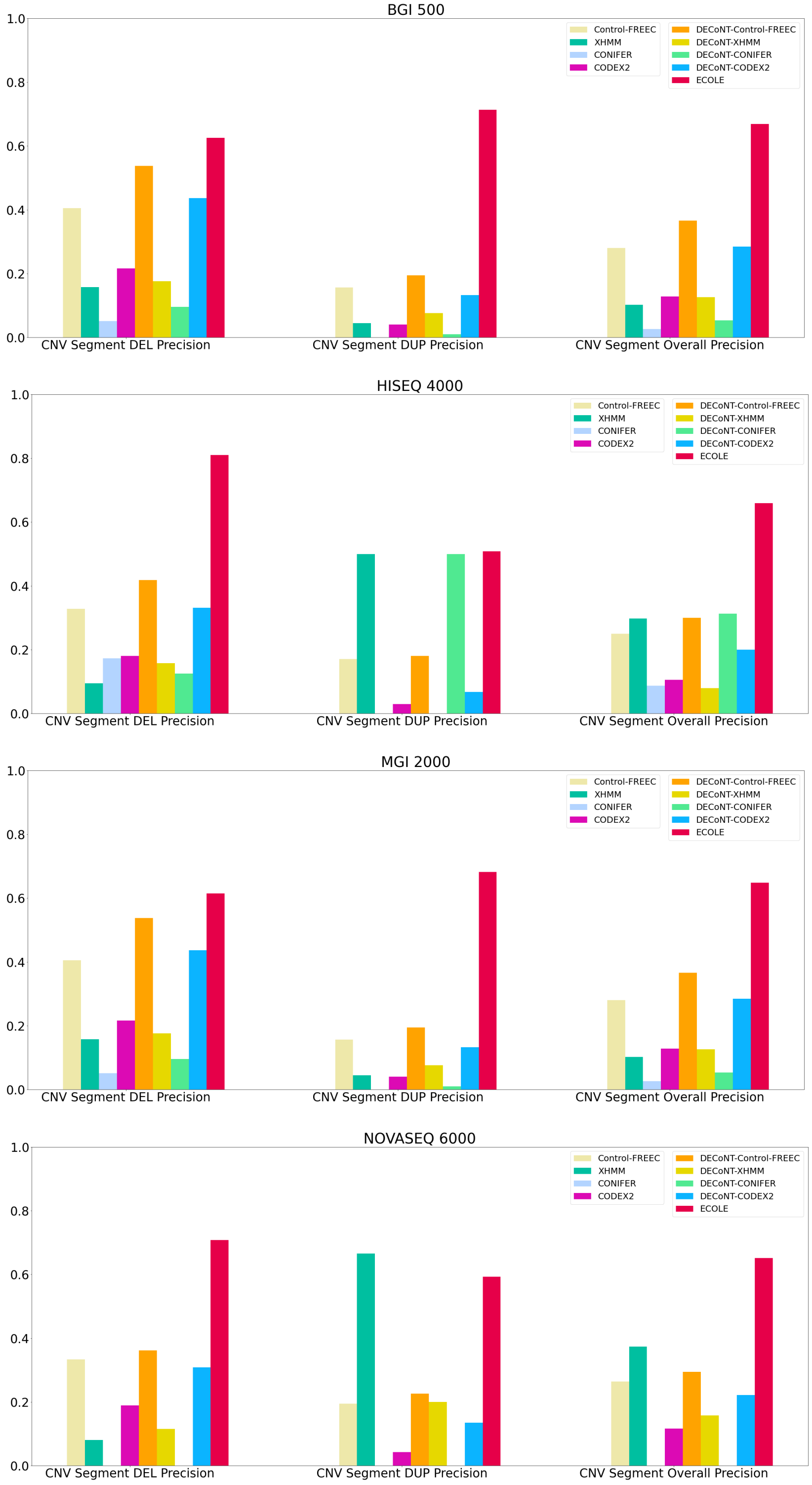

Supplementary Figure 18. Performance comparison with other tools on NA12878 sample on CNV segments. See Supplementary Table 24 for the values used to produce this plot and see Supplementary Tables 25-28 for the corresponding confusion matrices. Source data are provided as a Source Data file.

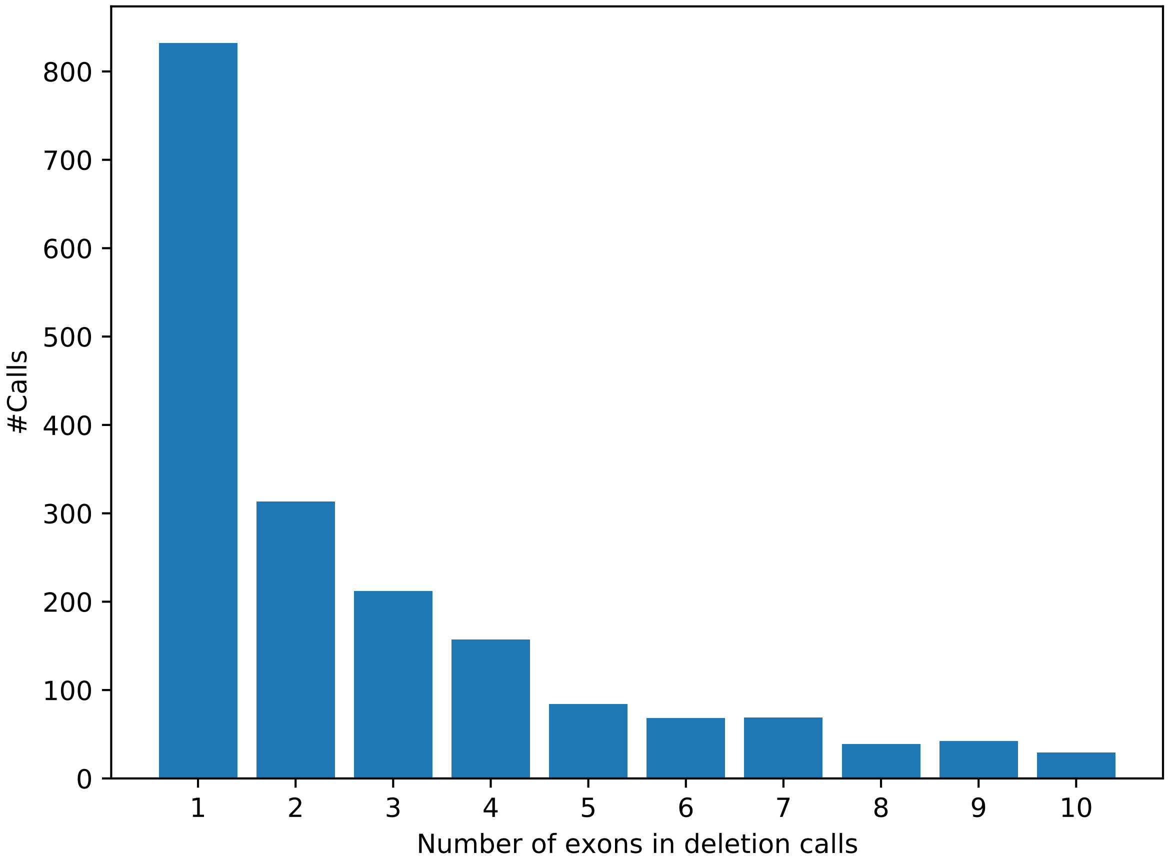

Supplementary Figure 19. Distribution of the number of exons in the merged deletion calls. Source data are provided as a Source Data file.

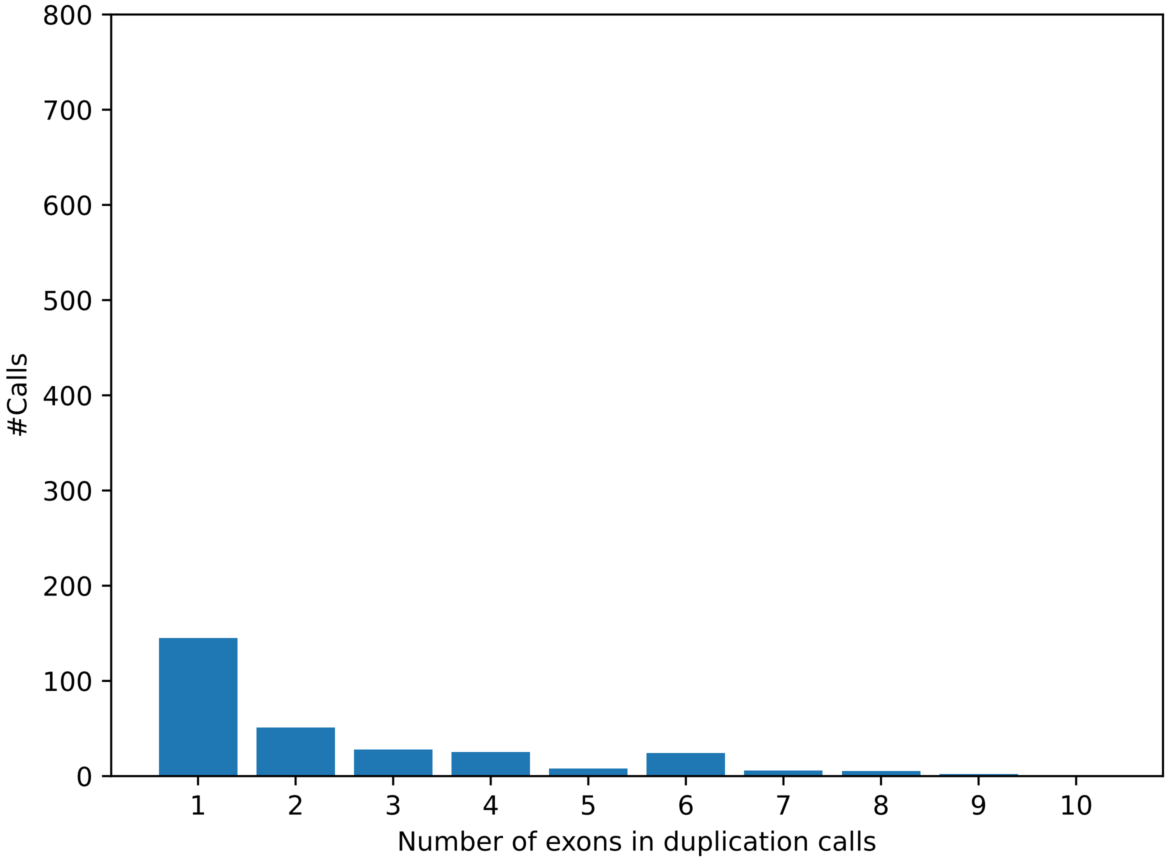

Supplementary Figure 20. Distribution of the number of exons in the merged duplication calls. Source data are provided as a Source Data file.

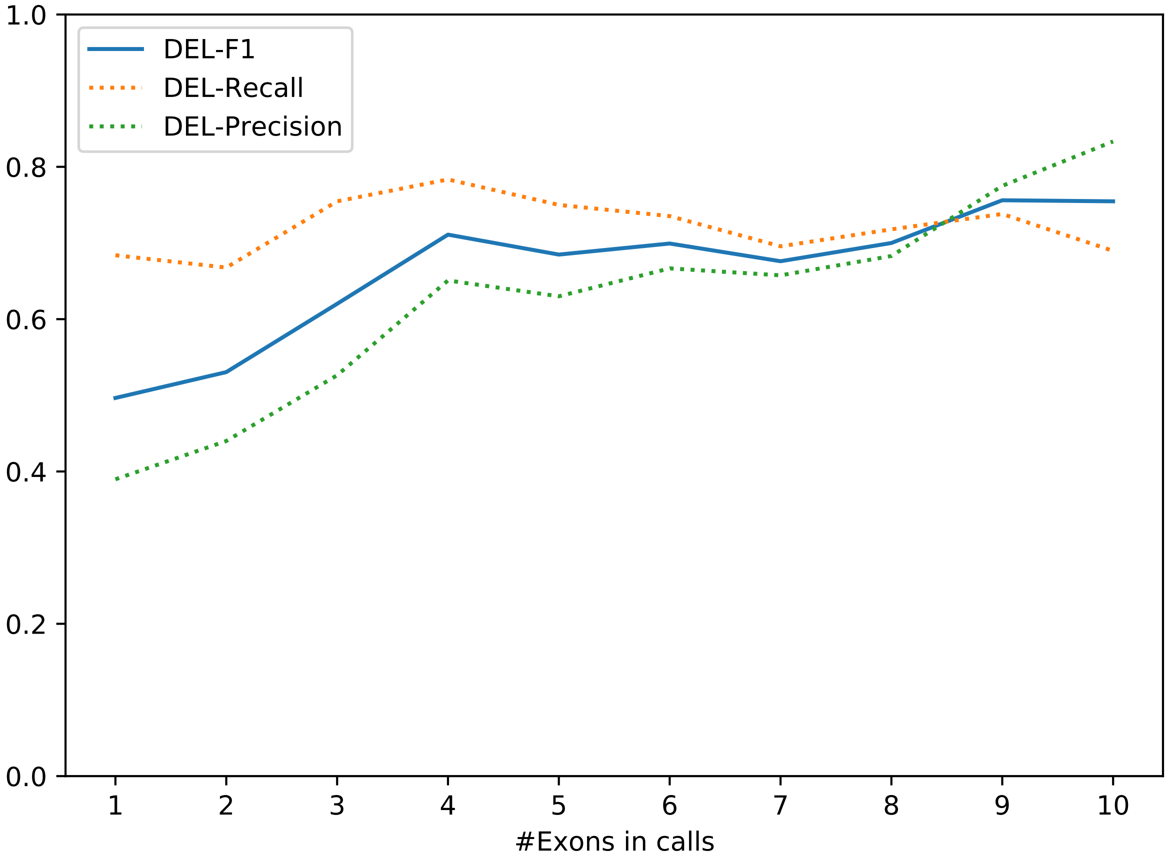

Supplementary Figure 21. Deletion performance (F1 score, Recall, Precision) of ECOLE by the number of exons in calls. Source data are provided as a Source Data file.

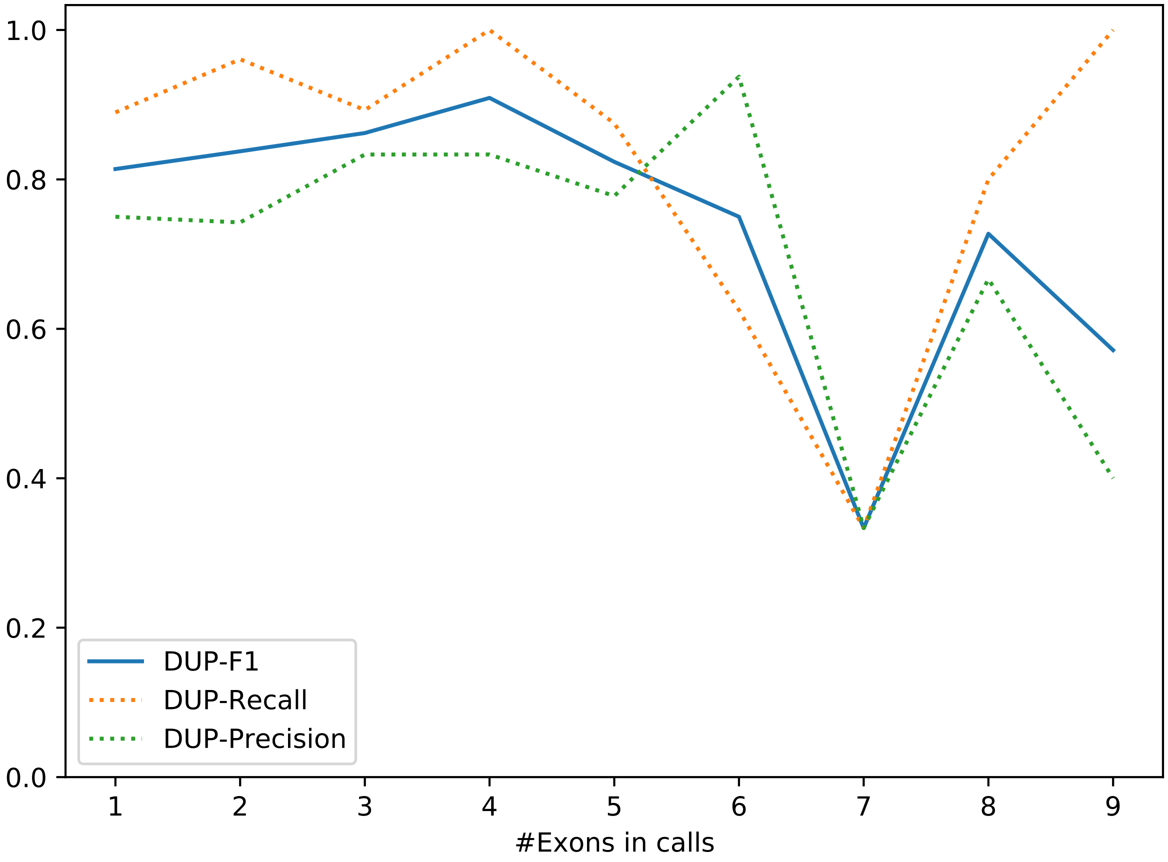

Supplementary Figure 22. Duplication performance (F1 score, Recall, Precision) of ECOLE by the number of exons in calls. Source data are provided as a Source Data file.

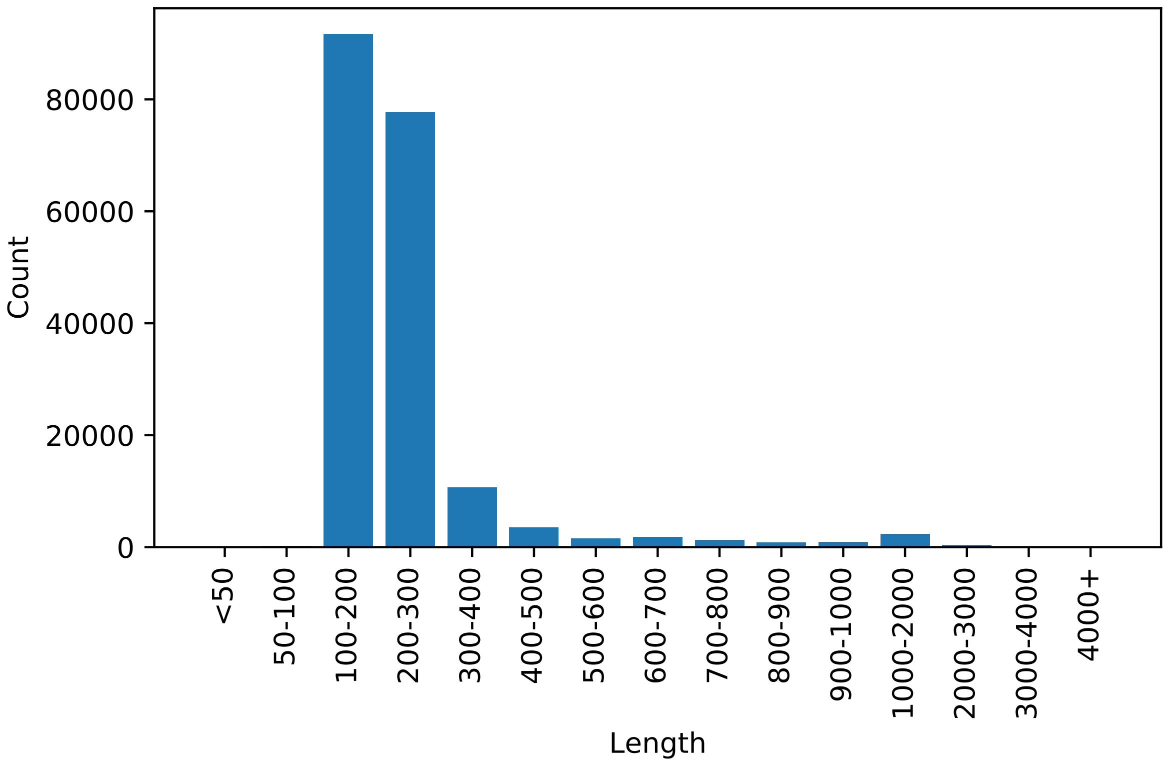

Supplementary Figure 23. The length distribution of exons. Source data are provided as a Source Data file.

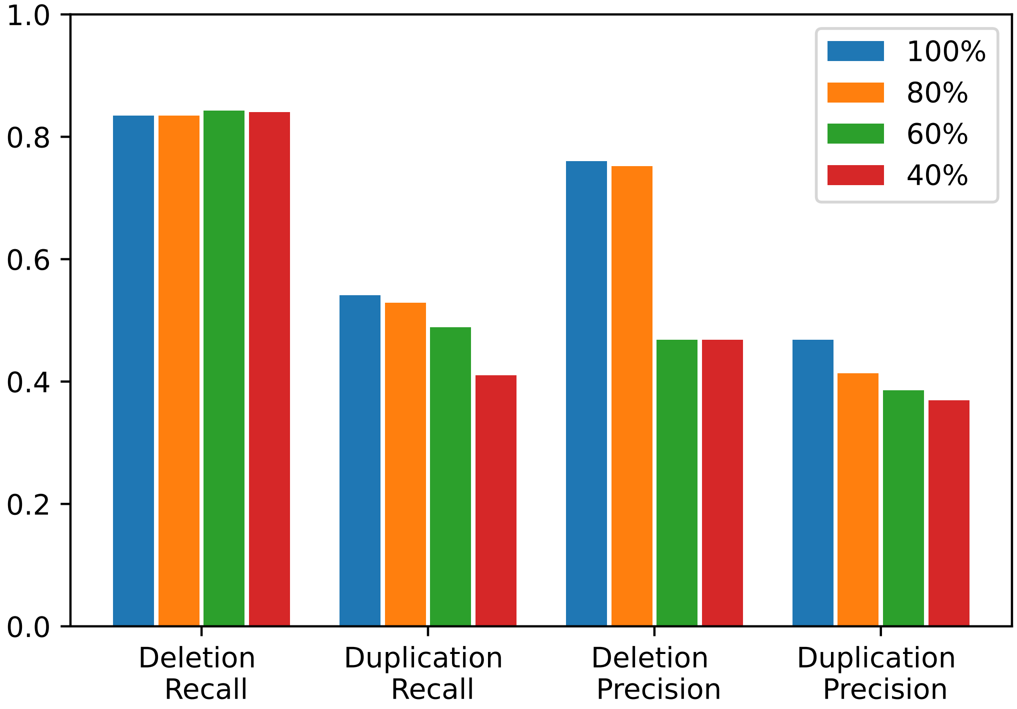

Supplementary Figure 24. The performance of ECOLE$^{\text{FT-GIAB}}$ on NA12892 samples subsampled at the rate of 80%, 60%, and 40%. Source data are provided as a Source Data file.

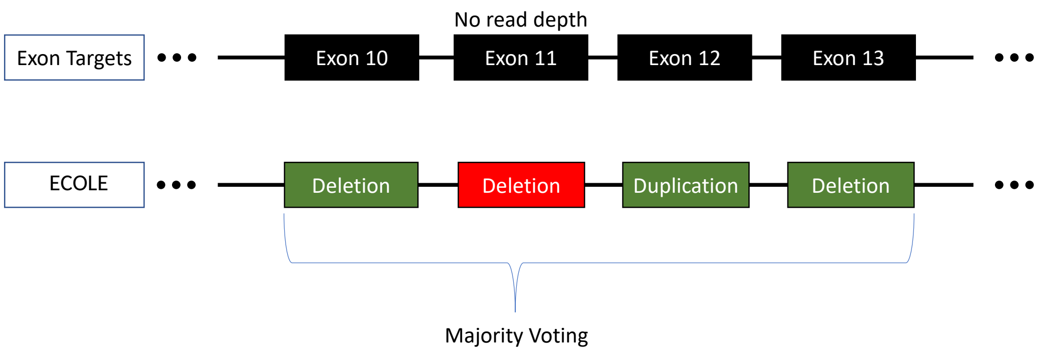

Supplementary Figure 25. Some exons do not have read depth information, thus ECOLE cannot make CNV calls for these exons immediately. Initially, ECOLE calls are obtained for exons that have read depth value. Later, the calls for exons that don't contain read depth information are obtained by applying majority voting on a window of size 3 closest exons on the ECOLE calls for neighboring exon regions. In this example, the red exon did not have a read depth signal and inferred using this method.

3 Supplementary Notes

3.1 Derivation of relevance scores for ECOLE interpretability

The attention mechanism introduced in [5] is as follows:

$$\mathbf{A} = softmax(\mathbf{Q} \cdot \mathbf{K}^) \tag{1}$$

$$\mathbf{O} = \mathbf{A} \cdot \mathbf{V} \tag{2}$$

where $\mathbf{O} \in R^{h \times s \times d_h}$ is the output of the multi-head attention, $\mathbf{Q} \in R^{h \times s \times d_h}$ is the query matrix, $\mathbf{K}, \mathbf{V} \in R^{h \times s \times d_h}$ are the key and value matrices. h is the number of heads in multi-head attention module, d_h is the size of the embedding dimension. $\mathbf{A}$ inherently defines the connections between each token along the sequence of length s. In order to fetch the relevance of the attention signals with respect to the classification, we will use the relevance scores defined in [2]. The relevancy scores define the importance of each token for the predicted class of the input. We initialize the relevance scores with an identity matrix as follows:

$$\mathbf{R} = I_s \tag{3}$$

where the identity matrix is $s \times s$ and s denotes sequence length. This explainability method uses the attention $\mathbf{A}$ matrix from each attention head and uses the gradient signals to average across the heads. The resulting attention map $\bar{\mathbf{A}} \in R^{s \times s}$, is as follows:

$$\bar{\mathbf{A}} = E_h((\nabla \mathbf{A} \odot \mathbf{A})^+) \tag{4}$$

where $\odot$ is the Hadamard product between the gradient matrix and the attention matrix $\nabla \mathbf{A} := \frac{\partial y_t}{\partial \mathbf{A}}$, and y_c is the prediction of the model for the class c we perform explanation, and E_h is average over the multiple attention heads.

$$\mathbf{R} = \mathbf{R} + \bar{\mathbf{A}} \cdot \mathbf{R} \tag{5}$$

We use the Attention matrices $\mathbf{A}$ of the last Transformer layer and obtain the specified relevance scores. Moreover, we are using Performer [3] with FAVOR+ mechanism which approximates self-attention and calculates the output without using the attention matrix explicitly. Therefore, we reconstruct the attention matrix explicitly by replacing the value matrix in Equation 2, with an identity matrix that contains the indices of the sequence tokens.

3.2 Hyperparameter Tuning for Baseline Models

First, we consider linear SVM classifier and XGBoost with the default hyperparameters. We trained these 2 baseline methods on our training set (1000 Genomes) for 150 rounds (partial fit). We then tested the trained methods using the 1000 Genomes test data set. The performance benchmark of these methods with default parameters and ECOLE can be seen in Supplementary Table 29.

To make these methods stronger baselines, we decide to tune the hyperparameters. For this, we adopt a 5-fold cross-validation routine and applied a grid search on the validation set to select the best hyperparameters for the linear SVM classifier and XGBoost. We selected the validation set for each batch of the training set in the current round. We set the validation set as 20% of the training set. We used a random seed for this selection. We then trained these 2 methods using the best hyperparameter setting for each batch and round of the training. Finally, we tested the trained methods with tuned hyperparameters. However, the SVM classifier and XGBoost with tuned hyperparameters performed similarly to the methods with default hyperparameters, Thus, we chose to include the default SVM classifier and XGBoost as our baseline model.

The hyperparameter search space for linear SVM classifier was as follows: (1) the loss function to be used $\in \{'hinge','log_loss','squared_error'\}$; (2) the penalty to be used $\in \{"l2","l1","elasticnet", None\}$; (3) constant that multiplies the regularization term sampled 5 equidistant learning rates from $[10^{-3}, 10^{-4}]$ and (4) the maximum number of passes over the training data $\in \{500, 1000, 2000\}$. The hyperparameter search space for XGBoost was as follows: (1) the loss function to be used $\in \{`log_loss', `exponential'\}$; (2) the function to measure the quality of a split. $\in \{`friedman_mse', `squared_error'\}$; (3) the minimum number of samples required to split an internal node $\in \{2, 5, 10\}$; (4) the minimum number of samples required to be at a leaf node $\in \{1, 2, 5\}$ and (5) maximum depth of the individual regression estimators $\in \{2, 3, 4\}$.

3.3 Chromosome-specific CNN model

This CNN-based model contains 4 layers of 1-dimensional convolutional neural network and 1 linear layer of 311 neurons. The kernel sizes of the 4 layers of CNN are as follows: 100, 100, 160, 160. We use ReLU as activation function. We use Max Pooling and Dropout between the last CNN and linear layer. We trained 2 different CNN models for chromosomes 21 and 10. In Table 30, the results of these 2 chromosome-specific CNN models along with ECOLE are shown. Note that we trained and tested these 2 chromosome-specific CNN-based models on the same respective chromosomes. On chromosome 21, even though the CNN-based model attains a high precision (58.8%), ECOLE is able to outperform this model in both precision (85.5%) and recall (92.6%). On chromosome 10, the CNN-based model performs very poorly and attains 0% precision and recall. On the other hand, ECOLE performs better than the CNN-based solution and attains 3.7% precision and 29.2% recall. We observe that CNN-based models perform much worse than ECOLE in their respective chromosomes.

3.4 The Architecture of Generic CNN model

The CNN model contains 5 layers in total - 4 convolutional layers and a fully connected layer. The first CNN layer is a 1D convolutional layer containing 100 kernels with a size of 10 with stride 1. It takes the input data R^{1024}, returns an output vector $\in R^{100x1015}$ followed by ReLU activation. The second convolutional layer uses 100 kernels with size 10. It generates the vector $\in R^{100x1006}$ again followed by ReLU activation and max pooling to, resulting in the output $\in R^{100x335}$. Subsequently, two more 1D convolutional layers are applied with kernel sizes of 160, and ReLU activation is performed after each convolutional layer. It returns a vector $\in R^{160x317}$. Then, global max-pooling is applied resulting in a 317 dimensional vector. To prevent overfitting, a dropout layer is used. Finally, a fully connected layer is used to map the output of the previous layer to the desired 3-dimensional output followed by softmax activation. Each dimension corresponds to labels: DEL, DUP, and no-call, respectively. We use the weighted cross-entropy loss function.

3.5 ECOLE trained with different maximum read depth length values

We train ECOLE models that input 100-, 200-, 500-, and 1000-sized input read depth vectors. We use the same hyperparameters discussed in Section 4.2 of the manuscript. We then test these models using our test samples. We observe that ECOLE trained with a 100-sized read depth vector performed best for calls smaller than 100bp with an overall F1-score of 31.4%. In contrast, ECOLE trained with the 1000-sized read depth vector achieved a 12.6% overall F1 score for these calls. We would like to note that the number of ground truth CNV calls for this region (<100bp) is very small (3253) compared to regions sized between 100-200 bp (970571). For calls that are between 100 bp and 200 bp in size, ECOLE trained with a 1000-sized read depth vector outperforms ECOLE models trained with 200-, and 500-sized read depth vectors with an overall F1-score of 61.5% (7.5% and 14.7% improvement, respectively). Note that the model trained with a 100-sized read depth vector is not applicable for this and larger size ranges. Finally, for calls longer than 200 bp, ECOLE trained with the 1000-sized read depth vector performs better than the version trained with a 500-sized read depth vector with an overall F1 score of 63.9% (15.6% improvement). While for very short exons, one can opt for the ECOLE model, which uses a 100-sized read depth vector, that model cannot make predictions for larger exons which is very restrictive. Since a 1000bp input vector covers most of the exons in the exome, and shows overall good performance, we provide results with this ECOLE version. Please see Supplementary Table 32 for the deletion and duplication prediction performances of these models.

Supplementary References

1. Chaisson, M.J., Sanders, A.D., Zhao, X., Malhotra, A., Porubsky, D., Rausch, T., Gardner, E.J., Rodriguez, O.L., Guo, L., Collins, R.L., et al.: Multi-platform discovery of haplotype-resolved structural variation in human genomes. Nature communications **10**(1), 1–16 (2019)
2. Chefer, H., Gur, S., Wolf, L.: Generic attention-model explainability for interpreting bi-modal and encoder-decoder transformers (2021)
3. Choromanski, K., Likhosherstov, V., Dohan, D., Song, X., Gane, A., Sarlos, T., Hawkins, P., Davis, J., Mohiuddin, A., Kaiser, L., Belanger, D., Colwell, L., Weller, A.: Rethinking attention with performers (2021)

4. Guo, G., Sun, X., Chen, C., Wu, S., Huang, P., Li, Z., Dean, M., Huang, Y., Jia, W., Zhou, Q., et al.: Whole-genome and whole-exome sequencing of bladder cancer identifies frequent alterations in genes involved in sister chromatid cohesion and segregation. Nature genetics **45**(12), 1459–1463 (2013)
5. Vaswani, A., Shazeer, N., Parmar, N., Uszkoreit, J., Jones, L., Gomez, A.N., Kaiser, L., Polosukhin, I.: Attention is all you need (2017)
