# Supplementary material for: ECOLE: Learning to call copy number variants on whole exome sequencing data
Source: Nat Commun. 2024 Jan 2;15:132. doi: 10.1038/s41467-023-44116-y (PMC10762021; doi:10.1038/s41467-023-44116-y)
Supplement: Supplementary file 3 — Description of Additional Supplementary Files [file 41467_2023_44116_MOESM3_ESM.pdf]

Description of Additional Supplementary Files:

Supplementary Dataset 1: Lists of samples used for training and testing ECOLE in different scenarios are given. This data is provided as a separate Excel file.
