# Supplementary material for: ECOLE: Learning to call copy number variants on whole exome sequencing data
Source: Nat Commun. 2024 Jan 2;15:132. doi: 10.1038/s41467-023-44116-y (PMC10762021; doi:10.1038/s41467-023-44116-y)
Supplement: Supplementary file 5 — Reporting Summary [file 41467_2023_44116_MOESM5_ESM.pdf]

nature portfolio

Corresponding author(s): A. Ercument Cicek

Last updated by author(s): Nov 3, 2023

Reporting Summary

Nature Portfolio wishes to improve the reproducibility of the work that we publish. This form provides structure for consistency and transparency in reporting. For further information on Nature Portfolio policies, see our Editorial Policies and the Editorial Policy Checklist.

Statistics

For all statistical analyses, confirm that the following items are present in the figure legend, table legend, main text, or Methods section.

n/a	Confirmed	
☐	☒	The exact sample size (*n*) for each experimental group/condition, given as a discrete number and unit of measurement
☐	☒	A statement on whether measurements were taken from distinct samples or whether the same sample was measured repeatedly
☒	☐	The statistical test(s) used AND whether they are one- or two-sided<br>*Only common tests should be described solely by name; describe more complex techniques in the Methods section.*
☒	☐	A description of all covariates tested
☒	☐	A description of any assumptions or corrections, such as tests of normality and adjustment for multiple comparisons
☐	☒	A full description of the statistical parameters including central tendency (e.g. means) or other basic estimates (e.g. regression coefficient) AND variation (e.g. standard deviation) or associated estimates of uncertainty (e.g. confidence intervals)
☒	☐	For null hypothesis testing, the test statistic (e.g. *F*, *t*, *r*) with confidence intervals, effect sizes, degrees of freedom and *P* value noted<br>*Give P values as exact values whenever suitable.*
☒	☐	For Bayesian analysis, information on the choice of priors and Markov chain Monte Carlo settings
☐	☒	For hierarchical and complex designs, identification of the appropriate level for tests and full reporting of outcomes
☒	☐	Estimates of effect sizes (e.g. Cohen's *d*, Pearson's *r*), indicating how they were calculated

Our web collection on statistics for biologists contains articles on many of the points above.

Software and code

Policy information about availability of computer code

Data collection	N/A
Data analysis	Read depth calculation of .bam files performed using sambamba tool v0.7.0. XHMM v1.0, CODEX2, CoNIFER (v0.2.2), Control-FREEC v11.5, GATK v4, CNLearn v1 and CNV-Kit v0.9.7 WES-CNV calling tools are used to produce the CNV calls. DECoNT is used to polish these calls. CNVnator v0.4.1 is used to get WGS calls, and WGS CNV calls are matched with WES CNV calls obtained from WES-based CNV callers using custom Python scripts. Scripts are available on the Zenodo repository with necessary instructions to train a model or to simply use an already trained model. https://zenodo.org/records/8202814

For manuscripts utilizing custom algorithms or software that are central to the research but not yet described in published literature, software must be made available to editors and reviewers. We strongly encourage code deposition in a community repository (e.g. GitHub). See the Nature Portfolio guidelines for submitting code & software for further information.

Data

Policy information about availability of data

All manuscripts must include a data availability statement. This statement should provide the following information, where applicable:

- Accession codes, unique identifiers, or web links for publicly available datasets
- A description of any restrictions on data availability
- For clinical datasets or third party data, please ensure that the statement adheres to our policy

All the data for reproduction is available under the license CC BY-NC-SA 2.0 at https://zenodo.org/record/8202814 (DOI: 10.5281/zenodo.8202814). Please note

that ECOLE software is completely free for academic usage. However, it is licensed for commercial usage. Source data are provided with this paper for reproducing all Figures in the manuscript and Supplementary Info. All datasets used in this study are publicly available. We provide links to all data sources in the Data Availability section. We also provide custom scripts, which let users to reproduce the results presented in all figures and tables in the main text. The necessary data and the scripts are deposited to Zenodo and the details are provided in the Data/Software Availability sections. The 1000 Genome Project sample names we used to train and test the models are provided in the Methods section which are available at the 1000 Genomes Project. WES and WGS samples are available at the following link: ftp://ftp.1000genomes.ebi.ac.uk/vol1/ftp/data_collections/1000_genomes_project/data/. Guo et al. samples are available at Sequence Reads Archive under the accession code SRP017787 [https://www.ncbi.nlm.nih.gov/sra/?term=SRP017787]. . GiaB CNV call sets for the Ashkenazi family are available under the accession codes SAMEA1573615 and SAMEA1573616 [https://ftp-trace.ncbi.nlm.nih.gov/giab/ftp/technical/svclassify_Manuscript/Supplementary_Information/metasv_trio_validation/]. The labels we use for training, fine-tuning, and testing are available at the following link: https://zenodo.org/record/8202814. The 1000 Genome Project labels, Guo et al., Chaisson et al. and Ashkenazi family labels can be accessed on the Zenodo repository.

Research involving human participants, their data, or biological material

Policy information about studies with human participants or human data. See also policy information about sex, gender (identity/presentation), and sexual orientation and race, ethnicity and racism.

Reporting on sex and gender	Sex and gender are not considered in this study. The sex and gender information have not been collected while obtaining the dataset.
Reporting on race, ethnicity, or other socially relevant groupings	Race, ethnicity and other groupings are not considered in this study. This information has not been collected while obtaining the dataset.
Population characteristics	We did not collect data for this study.
Recruitment	We did not recruit individuals for data collection.
Ethics oversight	N/A

Note that full information on the approval of the study protocol must also be provided in the manuscript.

Field-specific reporting

Please select the one below that is the best fit for your research. If you are not sure, read the appropriate sections before making your selection.

☒ Life sciences ☐ Behavioural & social sciences ☐ Ecological, evolutionary & environmental sciences

For a reference copy of the document with all sections, see nature.com/documents/nr-reporting-summary-flat.pdf

Life sciences study design

All studies must disclose on these points even when the disclosure is negative.

Sample size	We use aligned WES reads from 1000 Genomes Project. We select the first 1000 samples from the alphabetically ordered list of WGS samples (HG00096 to HG02356). We use 707 samples out of these 1000, for which matched WES samples are available.
Data exclusions	N/A
Replication	We reproduce our test results using highly validated WGS CNV call set presented in Chaisson et al., bladder cancer samples CNV set presented in Guo et al. and Ashkenazi Trio presented in GiaB Also we perform reproductions on WES CNV's of the sample NA12878 using reads obtained from four different sequencing platforms. The reproductions are successful.
Randomization	We randomly select 550 WES samples for training and 157 for testing.
Blinding	N/A

Reporting for specific materials, systems and methods

We require information from authors about some types of materials, experimental systems and methods used in many studies. Here, indicate whether each material, system or method listed is relevant to your study. If you are not sure if a list item applies to your research, read the appropriate section before selecting a response.

Materials & experimental systems

n/a	Involved in the study
x	Antibodies
x	Eukaryotic cell lines
x	Palaeontology and archaeology
x	Animals and other organisms
x	Clinical data
x	Dual use research of concern
x	Plants

Methods

n/a	Involved in the study
x	ChIP-seq
x	Flow cytometry
x	MRI-based neuroimaging
